# Supplementary material for: Utilisation of private general practitioners to provide caesarean deliveries in five rural district public hospitals in South Africa: a mixed-methods study
Source: BMJ Open. 2023 Mar 1;13(3):e067663. doi: 10.1136/bmjopen-2022-067663 (PMC9980375; doi:10.1136/bmjopen-2022-067663)
Supplement: Supplementary data [file bmjopen-2022-067663supp001.pdf]

Supplementary tables

Supplementary table 1: Summary of deliveries and private GP utilisation 01 April 2021 – 31 March 2022

	Hospital A		Hospital B		Hospital C		Hospital D		Hospital E		All Hospitals	
**Delivery profile**	**N**	**%**	**N**	**%**	**N**	**%**	**N**	**%**	**N**	**%**	**N**	**%**
Normal vaginal delivery (NVD)	311	75	356	80	732	74	1,259	85	1,271	74	3,929	78
Assisted vaginal (vacuum or forceps)	12	3	9	2	17	2	13	1	21	1	72	1
Caesarean deliveries	89	22	81	18	242	24	199	14	436	25	1,047	21
**Total**	**412**		**446**		**991**		**1,471**		**1,728**		**5,048**	
**Private GP utilisation for caesarean deliveries**												
**Surgeon**												
Public Sector non specialist Surgeon	0	0	1	1	148	61	152	76	394	90	706	67
Private GP Surgeon	89	100	80	99	94	39	47	24	42	10	341	33
**Anaesthetist**												
Public sector non specialist anaesthetist	0	0	81	100	63	26	148	74	436	100	746	71
Private GP Anaesthetist	89	100	0	0	179	74	51	26	0	0	300	29
**Profile of caesarean deliveries**												
**Hours, Monday-Sunday:**												
Evening/ night-time (4pm-8am)	49	55	52	64	103	43	88	44	150	34	442	42
Daytime hours (8am-4pm)	40	45	29	36	139	57	111	56	286	66	605	58
**Elective vs. Emergency**												
Elective caesarean	32	36	26	32	71	29	56	28	121	28	306	29
Emergency caesarean	57	64	55	68	171	71	143	72	316	72	742	71
**Anaesthetic Type**												
Spinal	86	97	79	99	218	92	192	96	414	95	989	95
General anaesthetic	3	3	1	1	16	7	5	3	20	4.5	45	4
Combined spinal and general	0	0	0	0	3	1	2	1	2	0.5	7	1

Supplementary table 2: Indications for emergency caesarean deliveries

Indications *	Hospital A		Hospital B		Hospital C		Hospital D		Hospital E		All Hospitals	
	N	%	N	%	N	%	N	%	N	%	N	%
Fetal distress/abnormal CTG	15	26	28	51	77	45	79	55	155	49	354	48
Prolonged labour/CPD	6	10	10	18	24	14	18	12	37	12	95	13
Previous CD (in labour)	4	7	3	5	42	25	9	6	12	4	70	9
Malpresentation (in labour)	12	21	6	11	11	6	11	8	30	10	70	9
Failed induction	2	4	1	2	2	1	8	6	27	8	40	5
placental abruption	2	4	1	2	5	3	6	4	3	1	17	2
Pre-eclampsia	0	0	1	2	0	0	4	3	9	3	14	2
PROM	3	5	0	0	0	0	0	0	3	1	6	1
Multiple pregnancy	1	2	2	4	0	0	2	1	0	0	5	1
APH	0	0	0	0	0	0	1	1	2	1	3	0.4
Failed VBAC	0	0	0	0	0	0	0	0	0	0	0	0
Big baby	0	0	0	0	0	0	0	0	0	0	0	0
Other	2	4	3	5	4	2	4	3	17	5	30	4
Number without listed indication	10	17	0	0	6	4	1	1	20	6	37	5
**Grand Total**	**57**	**100.00%**	**55**	**100.00%**	**171**	**100.00%**	**143**	**100.00%**	**315**	**100.00%**	**741**	**100.00%**

CTG = cardiotocography ; CPD= cephalopelvic disproportion; APH = antepartum haemorrhage; CD = caesarean delivery; VBAC = vaginal birth after Caesarean;

PROM = premature rupture of membranes; APH = antepartum haemorrhage

*The indications for caesarean deliveries were those that were written in the theatre register so it was not possible to interrogate some of the indications such as PROM (prolonged rupture of membranes). According to Western Cape guidelines, caesarean delivery can be performed at District hospitals for suspected Big Baby. However, VBACs are not routinely performed at District hospitals and patients with pre-eclampsia would all be referred to regional or tertiary hospitals

Supplementary table 3: indications for elective caesarean sections

Indications*	Hospital A		Hospital B		Hospital C		Hospital D		Hospital E		All Hospitals	
	N	%	N	%	N	%	N	%	N	%	N	%
Previous CD	5	16	16	61	50	70	40	71	66	55	177	58
Malpresentation (breech/transverse)	2	6	0	0	4	6	5	9	13	11	24	8
HIV & unsuppressed VL	1	3	2	8	0	0	0	0	8	7	11	3
Multiple pregnancy	0	0	0	0	1	1.4	1	2	3	2	5	2
Big baby	0	0	0	0	1	1.4	0	0	3	2	4	1
Patient request	0	0	0	0	0	0	0	0	2	2	2	1
Diabetes & macrosomia	0	0	0	0	0	0	0	0	1	1	1	0.3
Obstructive vulvar warts	0	0	0	0	0	0	0	0	0	0	0	0
Other (genital herpes etc)	1	3	1	4	7	10	1	2	10	8	20	7
Number without listed indication	23	72	7	27	8	11	9	16	15	12	62	20
**Grand Total**	**32**	**100.00%**	**26**	**100.00%**	**71**	**100.00%**	**56**	**100.00%**	**121**	**100.00%**	**306**	**100.00%**

CD = caesarean delivery; VL = viral load

*The indications for caesarean deliveries were those that were written in the theatre register so it was not possible to interrogate some of the indications. According to Western Cape guidelines, caesarean delivery can be performed at District hospitals for suspected Big Baby.

Supplementary table 4: Adverse outcomes following caesarean deliveries

	Hospital A		Hospital B		Hospital C		Hospital D		Hospital E		All Hospitals	
	**N**	**%**	**N**	**%**	**N**	**%**	**N**	**%**	**N**	**%**	**N**	**%**
**Adverse Outcomes**												
Total PPH/estimated blood loss >=1000 mls	0	0	1	1	2	1	0	0	10	2	13	1
Maternal deaths post caesarean	0	0	0	0	1*	0.4	0	0	0	0	1	0.1
Referrals post caesarean to a regional hospital	3	3	3	4	12	5	11	6	11	3	40	4
**Procedures performed at caesarean deliveries**												
IUCD/tubal ligation	13	15	5	6	33	14	16	8	43	10	110	11
Other Procedures	0	0	0	0	0	0	5	3	o	0	5	0.5
Hysterectomies at/after caesarean	0	0	0	0	0	0	0	0	0	0	0	0
B lynch suture/balloon tamponade	0	0	0	0	0	0	0	0	0	0	0	0
Relook laparotomies	0	0	0	0	0	0	0	0	0	0	0	0

PPH = postpartum haemorrhage; IUCD = intrauterine contraceptive device * cause of death was eclampsia
