# Supplementary material for: Utilisation of private general practitioners to provide caesarean deliveries in five rural district public hospitals in South Africa: a mixed-methods study
Source: BMJ Open. 2023 Mar 1;13(3):e067663. doi: 10.1136/bmjopen-2022-067663 (PMC9980375; doi:10.1136/bmjopen-2022-067663)
Supplement: Author's manuscript [file bmjopen-2022-067663.draft_revisions.pdf]

BMJ Open

BMJ Open is committed to open peer review. As part of this commitment we make the peer review history of every article we publish publicly available.

When an article is published we post the peer reviewers' comments and the authors' responses online. We also post the versions of the paper that were used during peer review. These are the versions that the peer review comments apply to.

The versions of the paper that follow are the versions that were submitted during the peer review process. They are not the versions of record or the final published versions. They should not be cited or distributed as the published version of this manuscript.

BMJ Open is an open access journal and the full, final, typeset and author-corrected version of record of the manuscript is available on our site with no access controls, subscription charges or pay-per-view fees (http://bmjopen.bmj.com).

If you have any questions on BMJ Open's open peer review process please email info.bmjopen@bmj.com

BMJ Open

# Utilisation of private general practitioners to provide caesarean deliveries, and maternal outcomes in five rural district public hospitals: Insights for public-private contracting

| | |
|---|---|
| Journal: | *BMJ Open* |
| Manuscript ID | bmjopen-2022-067663 |
| Article Type: | Original research |
| Date Submitted by the Author: | 23-Aug-2022 |
| Complete List of Authors: | Doherty, Tanya; South African Medical Research Council, Health Systems Research Unit<br>Solanki, Geetesh; South African Medical Research Council, HSRU<br>Daviaud, Emmanuelle; South African Medical Research Council<br>Bartmann, Yvandi; South African Medical Research Council<br>Hawkridge, Anthony; Western Cape Department of Health<br>Fawcus, Sue ; University of Cape Town Faculty of Health Sciences |
| Keywords: | OBSTETRICS, PUBLIC HEALTH, Organisation of health services < HEALTH SERVICES ADMINISTRATION & MANAGEMENT |
| | |

SCHOLARONE™
Manuscripts

*I, the Submitting Author has the right to grant and does grant on behalf of all authors of the Work (as defined in the below author licence), an exclusive licence and/or a non-exclusive licence for contributions from authors who are: i) UK Crown employees; ii) where BMJ has agreed a CC-BY licence shall apply, and/or iii) in accordance with the terms applicable for US Federal Government officers or employees acting as part of their official duties; on a worldwide, perpetual, irrevocable, royalty-free basis to BMJ Publishing Group Ltd ("BMJ") its licensees and where the relevant Journal is co-owned by BMJ to the co-owners of the Journal, to publish the Work in this journal and any other BMJ products and to exploit all rights, as set out in our licence.*

*The Submitting Author accepts and understands that any supply made under these terms is made by BMJ to the Submitting Author unless you are acting as an employee on behalf of your employer or a postgraduate student of an affiliated institution which is paying any applicable article publishing charge ("APC") for Open Access articles. Where the Submitting Author wishes to make the Work available on an Open Access basis (and intends to pay the relevant APC), the terms of reuse of such Open Access shall be governed by a Creative Commons licence – details of these licences and which Creative Commons licence will apply to this Work are set out in our licence referred to above.*

*Other than as permitted in any relevant BMJ Author's Self Archiving Policies, I confirm this Work has not been accepted for publication elsewhere, is not being considered for publication elsewhere and does not duplicate material already published. I confirm all authors consent to publication of this Work and authorise the granting of this licence.*

For peer review only

# Utilisation of private general practitioners to provide caesarean deliveries, and maternal outcomes in five rural district public hospitals: Insights for public-private contracting

Authors

Tanya Doherty[1,2], Geetesh Solanki[1], Emmanuelle Daviaud[1], Yvandi Bartmann[1], Anthony Hawkridge[3], Sue Fawcus[4]

Affiliations

[1] Health Systems Research Unit, South African Medical Research Council, Cape Town, South Africa

[2] Department of Paediatrics and Child Health, University of Cape Town, Cape Town, South Africa

[3] Swartland Subdistrict, Western Cape Department of Health, Malmesbury, South Africa

[4] Department of Obstetrics and Gynaecology, University of Cape Town, Cape Town, South Africa

**Correspondence to** Prof Tanya Doherty tanya.doherty@mrc.ac.za

For peer review only

## Abstract

### Objective

Researching how different models of maternity care in private and public facilities can work together to reduce inequities and promote universal access to safe obstetric care including caesarean delivery is essential. We aimed to document the utilisation of private general practitioners (GPs) contracted to provide caesarean delivery services in five rural district hospitals in the Western Cape.

### Methods

We utilised a mixed-method study design to describe rural district hospitals' utilization of private GP contracting for caesarean deliveries. Between April 2021 and March 2022 we collected routine data from delivery and theatre registers to capture the profile of deliveries and maternal outcomes. We conducted 27 semi-structured qualitative interviews with district managers, hospital employed doctors and midwives and private GPs to explore their experiences of the contracting arrangements.

### Results

The use of private GPs as surgeon or anaesthetist for caesarean deliveries differed widely across the hospitals. Overall, the utilisation of private GPs for anaesthetics was less (29% of all caesarean deliveries) than the utilisation of private GPs as surgeons (33% of all caesarean deliveries). The proportion of caesarean deliveries undertaken by private GPs as the primary surgeon showed a decreasing trend as size of hospital and total average monthly deliveries increased. Adverse outcomes following a caesarean delivery were rare. Qualitative data provided insights into contributions made by private GPs and the contracting models which did not incentivize over-servicing.

### Conclusion

The findings of this study suggest that private GPs can play an important role in filling "gaps" and expanding quality care in the public sector in areas where the total number of doctors or skills and expertise are in short supply, such as in rural areas. Different approaches to enable access to safe caesarean delivery are needed for different contexts and experienced private GPs are an under-appreciated public-private option for rural district hospitals.


For peer review only

**Key questions (3-5 sentences)**

**What is already known?**

- Strategies to promote universal access to safe obstetric care including caesarean delivery are urgently needed for low-and-middle income countries especially in rural contexts.
- Attempts to harness the resources of the private sector would need to address the challenge of ensuring that the care provided is appropriate and patterns of inappropriate care, such as high caesarean delivery rates, are not reproduced for the entire population.

**What are the new findings?**

- We found no trend towards an increased caesarean delivery rate in hospitals where a high proportion of caesarean deliveries were performed by private GPs compared to government employed generalist doctors and no increase in caesarean deliveries performed for elective indications.
- Our results suggest that private GPs can safely perform surgery and anaesthesia for caesarean deliveries in public district hospitals without compromising safety.

**What do the new findings imply?**

- Different approaches to enable access to safe caesarean delivery are needed for different contexts and experienced private GPs are an under-appreciated public-private option for rural district hospitals.

For peer review only

## Introduction

Maternal and child health is a major public health concern in South Africa (SA). Whilst there has been progress in reducing maternal mortality to 119 per 100 000 live births,[1] there remain huge disparities between public and private sectors in the distribution of obstetric care providers, models of care and outcomes.[2] In line with patterns seen in other low-and-middle-income countries,[3] the case fatality rate (CFR) for caesarean deliveries in the SA public sector is three times higher than for vaginal delivery and 27% of the caesarean delivery related CFR is associated with haemorrhage.[2] Improved clinical governance and skills development in the public sector are critical to reducing this disparity, but these efforts would need to be complemented by the mobilization of skills from the private sector given that the bulk of the resources lie in the private sector.[4]

SA is in the process of implementing major policy reform towards the achievement of Universal Health Coverage (UHC) through National Health Insurance (NHI). The NHI Bill[5] proposes that NHI will be a strategic purchaser of health care services for the entire SA population. The proposed NHI will contract for obstetric services from both public and private providers. A key challenge in SA regardless of when and in what form NHI is implemented, is to develop contracting models that allow public and private resources to be mobilized to the benefit of the entire population. This is particularly important for rural district hospitals where surgical skills and supervision may be insufficient to meet the service requirements for obstetric care, and specialist services are not readily available in the public or private sectors.

However, attempts to harness the resources of the private sector to service the needs of the broader SA population would need to address the challenge of ensuring that the care provided is appropriate and patterns of inappropriate care, the high caesarean delivery rate (74%) of the private sector for example[6], is not reproduced for the entire population. The development of contracting models that would best mitigate these risks is critical in delivering UHC.

Testing of different contracting models is urgently needed now, in the transition, and full implementation phases of NHI in SA and also to inform developments in other African countries that are implementing similar national health insurance financing arrangements. These include countries such as Ghana, Kenya, Nigeria and Tanzania.[7] There are some lessons to be learnt from public-private contracting for obstetric services in India where states have implemented different models of

For peer review only - http://bmjopen.bmj.com/site/about/guidelines.xhtml

contracting arrangements to increase access to obstetric care[8]. In some states private obstetricians are contracted in to public facilities to increase skills and human resources while in other states costs for services are subsidized within private facilities.[9]

Further research on how the different models of maternity care in private and public facilities can work together to reduce inequities and promote universal access to safe obstetric care including caesarean delivery is essential. Addressing these challenges in the area of obstetric care is particularly important because caesarean delivery is the most commonly performed surgical procedure and the findings may also be useful in informing strategies for other medical and surgical disciplines where similar patterns of under servicing and over-servicing in the public vs. private sector may occur.

We carried out research to document utilisation of private general practitioners (GPs) contracted to provide caesarean delivery services in five rural district hospitals in the Western Cape. In this paper we report on the extent of utilisation of private GPs, the profile of caesarean deliveries and maternal outcomes across the five hospitals; and examine the broader insights from the findings for public private contracting.

**Methods**

Study design

We undertook descriptive health systems research utilising a mixed-method study design to describe rural district hospitals' utilization of private GP contracting for caesarean deliveries in the Western Cape province and the profile and maternal outcomes of these deliveries in five hospitals. We chose a mixed-method study design incorporating both quantitative and qualitative data collection as this approach is well suited to applied health systems research.

Study setting

The setting for this research was the Western Cape province where existing public-private contracting for caesarean delivery services was occurring due to human resource shortages in rural district hospitals. Five rural district hospitals within one rural district were chosen following engagement with provincial managers and obstetric clinical managers.

For peer review only

In South Africa, district hospitals provide level 1 (generalist) services to in-patients and outpatients (ideally on referral from a community health center or clinic). District hospitals have between 30 and 200 beds, a 24-hour emergency service and an operating theatre. Generalists (medical officers) provide the services together with nursing staff and allied health professionals, some district hospitals have specialist family physicians serving as clinical managers but there are no obstetric or anaesthetic specialists at district hospital level. Most district hospitals also have community service doctors. These are doctors who have completed a two-year internship and are required to complete a further one year of community service.[10] For obstetric services at district hospital level normal vaginal deliveries are performed by midwives, assisted vaginal deliveries are performed by advanced midwives or medical officers and caesarean deliveries (surgery and anaesthesia) are performed by medical officers.

The public health service is permitted to contract the services of private providers where needed. There are three mechanisms by which private providers can be contracted to the public service: through a locum agency, through a sessional contract which is limited to a maximum of 39 hours per month or as a service provider in response to a tender for specific services. In the case of obstetric services private providers are mainly utilized for theatre services either as a GP surgeon or GP anaesthetist to undertake caesarean deliveries or for obstetric surgery including ectopic pregnancy, termination of pregnancy and dilatation and curettage following spontaneous miscarriage. They may also be called for an assisted delivery if the establishment doctor is unable to manage a complicated delivery. For GPs contracted through a sessional contract, medico-legal indemnity is provided by the state but for those contracted as locums or through a service provider tender they are required to have their own medico-legal indemnity cover.

Quantitative data collection

For a period of 12 months (1 April 2021 – 31 March 2022) we collected quantitative obstetric clinical data from all five participating hospitals. Clinical outcomes were captured from the delivery register, theatre register and obstetric transfer book and included: mode of delivery, indication for caesarean delivery and rates of caesarean delivery complications (maternal death, postpartum haemorrhage and referral to a regional hospital). Other procedures performed at or after a caesarean delivery were also captured as a measure of complications (hysterectomy, B-lynch suture and re-look laparotomy). For each caesarean delivery we captured whether the surgeon and anaesthetist was a hospital employed medical officer or a private GP, the type of anaesthetic given, whether the procedure was classified as

For peer review only

an emergency or elective and whether it was performed during working hours (8-4pm, Monday-Sunday) or after hours.

Clinical data was collected by research nurses recruited for the purpose of the study. The monthly data was entered into a pre-set excel spreadsheet.

Qualitative data collection

Semi-structured interview guides were developed, one for private providers and one for public providers. Qualitative interviews were undertaken by three of the investigators (TD, GS and ED) at the hospitals and district office.

Interviews were undertaken with private GPs who had entered into public service contracts with each of the five hospitals to explore their perceptions of undertaking work in the public sector. At each hospital we also interviewed the hospital managers and public providers (doctors and midwives) to explore their perceptions of the public/ private interface including contracting procedures, team work and clinical governance. During interviews we also collected information on the total number of hours per month each GP was contracted to the public hospital.

We undertook a total of 27 semi-structured qualitative interviews; including 3 district managers (2 male, 1 female), twelve private GPs (7 male, 5 female), 8 government employed doctors (4 male, 4 female) and 4 hospital midwives (all female).

Data analysis

Quantitative data from hospital records was analysed in excel using simple descriptive means and frequencies of annual deliveries, caesarean delivery rates, profile and outcomes stratifying by hospital.

We describe the proportion of total annual caesarean deliveries at each hospital undertaken by private GPs as surgeon or anaesthetist and explore the relationship between type of provider and the caesarean delivery profile (elective vs emergency and timing in terms of in hours or after hours) and adverse outcomes (referral to a regional hospital following a caesarean delivery).

Qualitative interviews were digitally recorded and transcribed. A framework analysis[11] approach was applied which is well suited to implementation research designs. We drew on a framework previously

For peer review only

developed by members of our research team which outlines drivers, challenges and required action for obstetric care in preparing for NHI.[12] The interviews were read and re-read by members of the team to familiarise ourselves with the content. Emerging categories and major themes were identified and mapped against the framework and quantitative findings. The qualitative data enhance contextual understanding of the quantitative outcomes related to CS rates and cost by shedding light on provider experiences and perceptions of aspects such as workload, supervision and remuneration.

Ethical considerations

Ethics approval was granted by the South African Medical Research Council Human Research Ethics Committee (approval number EC003-2/2021) and permission was also granted by the Western Cape Provincial Department of Health and Wellness (approval number WC_202103_017). No personal identifying information was captured from hospital records, only aggregated summary information. All health professionals who were approached to participate in an interview were read an information sheet outlining the expectations in terms of length of the discussion, the voluntary nature of the participation and measures to ensure confidentiality. Health professionals who agreed to participate signed an informed consent form.

**Patient and public involvement** Patients and/or the public were not involved in the design, or conduct, of this research. We will be disseminating the findings including through presentations at each of the participating hospitals which will be targeted at stakeholder groups including patients and members of the public.

## Results

### Hospital characteristics and utilisation of private GPs for caesarean deliveries

The five district hospitals ranged in size from 35 beds in hospital A to 118 beds in hospital E. Over the period 1 April 2021 to 31 March 2022, the average number of monthly deliveries ranged from 34 in hospital A to 144 in hospital E. None of the hospitals has primary care clinics in its catchment areas that perform vaginal deliveries other than in the case of emergencies and only during daytime hours. All five hospitals have some involvement of private GPs to assist with the obstetric service but the level of utilisation of their services differed greatly across the hospitals. In hospital A there are no government employed doctors and the hospital is run entirely by a private GP practice consisting of 5 doctors who

For peer review only

provide 24-hour cover to the hospital. Across the other hospitals the number of contracted GPs ranged from 1 in hospital E to 5 in hospital C but this translated into a maximum of 2 full time equivalent doctors in hospital C and 0.2 in hospital E (table 1).

For peer review only

Table 1: Characteristics of participating hospitals

| Hospital | Hospital A | Hospital B | Hospital C | Hospital D | Hospital E |
|---|---|---|---|---|---|
| Number of beds | 35 | 50 | 75 | 85 | 118 |
| Number of maternity beds (antenatal, labour and postnatal) | 8 | 10 | 12 | 14 | 25 |
| Number of clinics in the hospital catchment that perform vaginal deliveries | 0 | 5 clinics that can do emergency vaginal deliveries if required, no 24 hour service | 0 | 1 community day clinic, 4 clinics and 6 satellite services, none offer 24 hour service, can do emergency vaginal deliveries if required | 10 clinics that can do emergency vaginal deliveries if required, no 24 hour service |
| Monthly average number of deliveries | 34 | 37 | 83 | 123 | 144 |
| Number of fulltime hospital employed doctors | 0 | 6 | 6 | 10 (2 of which are specialist family physicians) | 13 |
| Number of community service doctors included in the total medical staff | 0 | 4 | 2 | 4 | 5 |
| Number of privately contracted GPs to assist with the obstetric service | One practice consisting of 5 GPs providing a 24 hour service | 2 | 5 (2 for anaesthetics and 3 for surgery) | 4 (2 surgical, 2 anaesthetic); 1 GP has a post-graduate diploma in anaesthetics, 1 GP has a post-graduate diploma in obstetrics, 1 GP is a specialist family physician | 1 |
| Number of full-time equivalent private GPs (based on a public sector doctor contract of 40 hours per week plus 16 hours overtime) | 0.9 | 0.3 | 2 | 0.9 | 0.2 |

Table 2 shows the profile of deliveries occurring in the hospitals over the 12-month study period. The proportion of vaginal unassisted deliveries ranged from 74% in hospitals C and E to 85% in hospital D. Assisted vaginal deliveries were uncommon (<3%) across all five hospitals. The caesarean delivery rate ranged from 14% in hospital D to 25% in hospital E. Spinal anaesthesia was used in 95% of all cases.

The use of private GPs as surgeon or anaesthetist for caesarean deliveries differed widely across the five hospitals. Overall, the utilisation of private GPs for anaesthetics was less (29% of all caesarean deliveries) than the utilisation of private GPs as surgeons (33% of all caesarean deliveries) although in hospital C more private GPs were utilised for anaesthetics than for surgery (Table 2). Interviews with both private and hospital employed doctors revealed that the skills shortage is mainly with surgical rather than anaesthetic skills, and that private GPs make a large contribution to training community service doctors in caesarean deliveries as these doctors explained:

*I'm doing more the cutting side. So, I mean, the comserves (community service doctor) can't cut. I am always cutting. There's one comserve on and it's me. So, we cut with the sister (Private GP, Hospital B).*

*So, in a month I must train all the community service doctors to be safe to do caesarean deliveries and ectopics which is not an easy undertaking (Clinical manager, Hospital D).*

*That is just the GP that is usually the primary surgeon. I am more of the assistant. I'm still learning to do caesars on my own. So, it's usually just the GPs who are doing the primary surgery. On the anaesthetic side there's another GP that is on call. We're basically an assistant. I'm still learning so I mostly doing primi-gravida's at the moment (Year 1 medical officer, Hospital C).*

*I'm on call every second day of my life and every second weekend of my life because we, as the two of us (private GPs), are the only people that can do caesarean deliveries. The rest of the people are either young MOs with little experience or, community service doctors that cannot do that (Private GP, Hospital B).*

For peer review only

Table 2: Summary of deliveries and private GP utilisation 01 April 2021 – 31 March 2022

| | Hospital A | | Hospital B | | Hospital C | | Hospital D | | Hospital E | | All Hospitals | |
|---|---|---|---|---|---|---|---|---|---|---|---|---|
| **Delivery profile** | **N** | **%** | **N** | **%** | **N** | **%** | **N** | **%** | **N** | **%** | **N** | **%** |
| Vaginal (unassisted) | 311 | 75 | 356 | 80 | 732 | 74 | 1,259 | 85 | 1,271 | 74 | 3,929 | 78 |
| Assisted vaginal (vacuum or forceps) | 12 | 3 | 9 | 2 | 17 | 2 | 13 | 1 | 21 | 1 | 72 | 1 |
| Caesarean deliveries | 89 | 22 | 81 | 18 | 242 | 24 | 199 | 14 | 436 | 25 | 1,047 | 21 |
| **Total** | **412** | | **446** | | **991** | | **1,471** | | **1,728** | | **5,048** | |
| **Private GP utilisation for caesarean deliveries** | | | | | | | | | | | | |
| **Surgeon** | | | | | | | | | | | | |
| Public Sector non specialist Surgeon | 0 | 0 | 1 | 1 | 148 | 61 | 152 | 76 | 394 | 90 | 706 | 67 |
| Private GP Surgeon | 89 | 100 | 80 | 99 | 94 | 39 | 47 | 24 | 42 | 10 | 341 | 33 |
| **Anaesthetist** | | | | | | | | | | | | |
| Public sector non specialist anaesthetist | 0 | 0 | 81 | 100 | 63 | 26 | 148 | 74 | 436 | 100 | 746 | 71 |
| Private GP Anaesthetist | 89 | 100 | 0 | 0 | 179 | 74 | 51 | 26 | 0 | 0 | 300 | 29 |
| **Profile of caesarean deliveries** | | | | | | | | | | | | |
| **Hours, Monday-Sunday:** | | | | | | | | | | | | |
| After hours (4pm-8am) | 49 | 55 | 52 | 64 | 103 | 43 | 88 | 44 | 150 | 34 | 442 | 42 |
| In hours (8am-4pm) | 40 | 45 | 29 | 36 | 139 | 57 | 111 | 56 | 286 | 66 | 605 | 58 |
| **Elective vs. Emergency** | | | | | | | | | | | | |
| Elective caesarean | 32 | 36 | 26 | 32 | 71 | 29 | 56 | 28 | 121 | 28 | 306 | 29 |
| Emergency caesarean | 57 | 64 | 55 | 68 | 171 | 71 | 143 | 72 | 316 | 72 | 742 | 71 |
| **Anaesthetic Type** | | | | | | | | | | | | |
| Spinal | 86 | 97 | 79 | 99 | 218 | 92 | 192 | 96 | 414 | 95 | 989 | 95 |
| General anaesthetic | 3 | 3 | 1 | 1 | 16 | 7 | 5 | 3 | 20 | 4.5 | 45 | 4 |
| Combined spinal and general | 0 | 0 | 0 | 0 | 3 | 1 | 2 | 1 | 2 | 0.5 | 7 | 1 |

For peer review only - http://bmjopen.bmj.com/site/about/guidelines.xhtml

The proportion of caesarean deliveries undertaken by private GPs as the primary surgeon showed a decreasing trend as size of hospital and total average monthly deliveries increased (figure 1). The two smallest hospitals were most reliant on private GPs to perform caesarean deliveries and as size of hospital increased fewer caesarean deliveries were undertaken by private GPs. The caesarean delivery rate was not higher in hospitals where private GPs carried out a higher proportion of caesarean procedures (figure 1). During interviews the reasons given for this were that it is generally the hospital-based staff who make the decision about whether a caesarean delivery is required, and the private GP is not involved in that decision making as these doctors described:

*The contract that I have is explicitly for theatre work only. Nothing else. I don't go into labour ward, maternity ward. I get called and I come straight to theatre. The decisions have been made by the medical officers in the ward or in casualty and then I'm the technician. I get called in to do mostly caesars but also ectopic pregnancies and incomplete abortions and that sort of thing. And complications of pregnancy, cervical tears, vaginal tears that sort of thing, retained placentas, but it's only theatre work (Private GP, Hospital E).*

*They book them. We don't really see the patient. We do the surgeries for them just in terms of logistics, and then they, they follow up with the aftercare after that (Private GP, Hospital C).*

**Profile and outcomes of caesarean deliveries with private GP contracting**

The majority (71%) of caesarean deliveries across all five hospitals were emergency procedures (table 2). The most common indication for an emergency caesarean delivery was foetal distress or an abnormal CTG (48%) (supplementary table 1). Amongst elective caesarean deliveries the main indication was a previous caesarean delivery (58%). For 5% of emergency caesarean deliveries and 20% of elective caesarean deliveries the indication was missing from the theatre register. Individual hospital documentation style differed with a higher proportion of elective caesarean deliveries (72%) with a documented indication missing at hospital A, which was serviced only by private GPs, whilst this was less than 30% at the other four hospitals. However, this did not affect the overall CS rate (supplementary table 2).

Adverse outcomes following a caesarean delivery indicated by blood loss >1000mls, maternal death post caesarean delivery and referral after caesarean delivery to a regional hospital were rare across all hospitals with a 1% prevalence of postpartum haemorrhage and 4% of women being transferred to a regional hospital following caesarean delivery. Over the 12-month period of data collection there were

For peer review only

no emergency procedures (hysterectomy, B-lynch suture or re-look laparotomies) performed during or following a caesarean delivery at any of the hospitals (table 3).

Figure 2 shows the utilisation of private GPs for caesarean deliveries and the profile and outcomes of these deliveries. Slightly over half of all caesarean deliveries were performed during public sector working hours (8am-4pm) but this differed across the hospitals. In hospitals A and B the majority of caesarean deliveries were performed after hours (4pm – 8am). In these two hospitals almost all of the caesarean deliveries are performed by private GPs who consult in their private practices during the day. Their time availability for hospital work is therefore more restricted in day-time hours as these participants explained:

*So, we come to hospital from eight to ten in the morning. Sometimes it does take longer and you only get down there at half past ten. So, from ten to twelve it's like private practice, then clinics, and then from half past two to half past four, five-ish at the practice again* (private GP, Hospital B).

*If it was a GP that was covering, they would be a bit reluctant to come in at four to assist us because having their own practice, they might still have practice till five or maybe six sometimes. So, they would maybe push the hour that they would be available to come in, a little bit further on, but, usually, if we call for a caesar, it's usually a foetal distress, we don't just cut to cut if it's not a foetal distress then we would say we could wait another half an hour (medical officer, hospital E).*

There is no noticeable increase in elective caesarean deliveries with a higher proportion of total caesarean deliveries undertaken by private GPs and rates of referral to a regional hospital following caesarean delivery was low irrespective of the utilisation of private GPs.

The funding models used to remunerate private GPs for their work within the public sector is a rate per shift irrespective of the number or type of procedures performed and therefore there is no undue incentive to perform unnecessary elective caesarean deliveries. Several GPs described the funding model during interviews:

*I do one week per month from Monday night, four p.m. till eight a.m. the next Monday and the remuneration is not affected by the number of call outs. It's a flat rate. Whether I get called once in that week or twenty times in that week, the remuneration is the same. Payment every month exactly the same. If I'm lucky, I get called out only once or twice for the week, but, a week ago, I was called out three times in one night. An ectopic pregnancy, abruptio and, obstructed labour. Just like that. It's four hours in theatre (private GP, Hospital E).*

For peer review only - http://bmjopen.bmj.com/site/about/guidelines.xhtml

*You never know how your hours are going to turn out for that week. So, say this week I have a very good week, I only have to be there from eight till eleven. They never phoned me during the night; they never phoned me during my day, the guy who's on next week they're phoning the whole time because it's a caesarean after caesarean. Every night there is an ectopic or a third-degree tear or something so you can't really, you can't really compare the two calls, but everyone still gets the same fixed rate (Private GP, Hospital C).*

*Because the sessional guys (private GPs) are just for the sessions. So, I mean they don't get paid extra if they do ten or one (caesarean deliveries) (Clinical manager, hospital D).*

For peer review only

Table 3: Adverse outcomes following caesarean deliveries

| | Hospital A | | Hospital B | | Hospital C | | Hospital D | | Hospital E | | All Hospitals | |
|---|---|---|---|---|---|---|---|---|---|---|---|---|
| | N | % | N | % | N | % | N | % | N | % | N | % |
| **Adverse Outcomes** | | | | | | | | | | | | |
| Total PPH/estimated blood loss >=1000 mls | 0 | 0 | 1 | 1 | 2 | 1 | 0 | 0 | 10 | 2 | 13 | 1 |
| Maternal deaths post caesarean | 0 | 0 | 0 | 0 | 1 | 0.4 | 0 | 0 | 0 | 0 | 1 | 0.1 |
| Referrals post caesarean to a regional hospital | 3 | 3 | 3 | 4 | 12 | 5 | 11 | 6 | 11 | 3 | 40 | 4 |
| **Procedures performed at caesarean deliveries** | | | | | | | | | | | | |
| IUCD/tubal ligation | 13 | 15 | 5 | 6 | 33 | 14 | 16 | 8 | 43 | 10 | 110 | 11 |
| Other Procedures | 0 | 0 | 0 | 0 | 0 | 0 | 5 | 3 | o | 0 | 5 | 0.5 |
| Hysterectomies at/after caesarean | 0 | 0 | 0 | 0 | 0 | 0 | 0 | 0 | 0 | 0 | 0 | 0 |
| B lynch suture/balloon tamponade | 0 | 0 | 0 | 0 | 0 | 0 | 0 | 0 | 0 | 0 | 0 | 0 |
| Relook laparotomies | 0 | 0 | 0 | 0 | 0 | 0 | 0 | 0 | 0 | 0 | 0 | 0 |

For peer review only

**Discussion**

This health systems research study explored the utilisation of private GPs to provide caesarean delivery services in rural district hospitals in the Western Cape. We found that the extent of utilisation of private GPs was inversely related to the size of hospital and number of deliveries. The two larger hospitals with over 100 monthly deliveries had a critical mass of doctors to cover the roster and used private GPs less frequently.

In terms of the delivery profile, we found an overall caesarean delivery rate of 21%. This is higher than the WHO recommended rate of 5-15% of all births[13] but lower than the public sector rate for the Western Cape of 29%.[2] This would be expected for district hospitals which have fewer high risk pregnancies compared to regional hospitals. We found no trend towards an increased caesarean delivery rate in hospitals where a high proportion of caesarean deliveries were performed by private GPs compared to government employed generalist doctors and no increase in caesarean deliveries performed for elective indications.

The public-private partnership arrangements in these hospitals are such that the decision-making regarding the need for a caesarean delivery is undertaken by a hospital employee (doctor or midwife) and the private GP is notified once the decision has been made. However, we did find a higher proportion of elective caesarean deliveries without stated indication and a higher proportion performed after hours at hospitals more reliant on private GPs, but this did not affect the overall caesarean delivery rate. The remuneration model for GP contracting, with a fixed rate irrespective of number of caesarean deliveries also prevents over-servicing. A public-private partnership programme in Gujarat state, India provides private obstetricians with a fixed sum per 100 births to provide care to eligible women below the poverty line. The remuneration model was specifically chosen as a disincentive to prevent unnecessary caesarean deliveries.[14] By comparison in Chile, where women covered under public health insurance can choose to give birth within a selected group of private health facilities the caesarean delivery rate was found to be 71% compared to 26% amongst publicly insured women giving birth in public health facilities. Publicly insured women choosing to give birth in a private facility receive a voucher that covers the costs of the delivery except for a 25% co-payment, the sum of which is dependent on the mode of delivery, which is the responsibility of the patient.[15]

In terms of caesarean delivery outcomes our results suggest that private GPs can safely perform caesarean deliveries in public district hospitals without compromising safety. There was one maternal death following caesarean delivery in the one-year period of review, a 1% rate of post-partum

For peer review only

haemorrhage and a very low rate of referral to a regional hospital following caesarean delivery. In SA, district hospitals account for 38% of public sector caesarean deliveries and the case fatality rate for caesarean deliveries at this level of care is 62 per 100,000 caesarean deliveries.[2] Since these hospitals do not have specialist obstetricians or anaesthetists, surgical and anaesthetic skills training and supervision is critical. Deaths due to bleeding at or following a caesarean delivery have been increasing in SA and lack of skilled doctors has been identified as an important avoidable factor.[16] This model of a public-private partnership makes an important contribution to skills development in rural district hospitals through drawing on the skills and experience of private GPs, and in some hospitals private family physicians, to perform caesarean deliveries alongside hospital-based medical officers many of whom are undertaking community service or recently qualified.

Balancing access with safety is a critical factor in obstetric care. Ideally facilities that can provide care for uncomplicated births should be close to women's homes and within reasonable distance of a district hospital that can perform caesarean deliveries. For small district hospitals with a low monthly delivery rate, maintaining skills to deal with emergencies is a challenge. To be safe and cost-effective, it has been recommended that district hospitals would need to perform between 500 and 1200 deliveries per year.[17] There should also always be a minimum of two doctors on call (one for anaesthesia and one for surgery), so that caesarean deliveries may be performed on a 24-hours-a-day, 7-days-a-week basis.[17] Hospitals A and B in this study performed less than 500 deliveries per year and had the highest utilisation of private GPs. It would be more costly for these hospitals to employ permanent doctors on the establishment and this model of public private contracting makes financial sense and provides access for women since the nearest regional hospital is roughly 150km from the district.

Considering broader implications of these findings for universal health care it is clear that experienced private GPs can play an important role in contributing much needed surgical and anaesthetic skills for safe caesarean deliveries in rural district hospitals. This is a model that could be considered for other provinces in SA and other low-and-middle income countries provided clinical decisions are driven by accepted public sector protocols and available support from specialists at referral regional hospitals can be ensured. Public-private partnerships have been criticized for being a more urban context option, but this is often on the assumption that the private providers will be obstetric specialists and this is the model that has been applied in settings such as India.[8 9 18] By contrast, in African countries such as Mozambique and Tanzania, clinical officers (healthcare providers trained to perform tasks usually undertaken by doctors) have been trained to perform caesarean deliveries. A meta-analysis reported no

For peer review only

significant differences in outcomes of caesarean deliveries performed by clinical officers compared to medical doctors.[19] Different approaches to enable access to safe caesarean delivery are therefore needed for different contexts and experienced private GPs are an under-appreciated public-private option for rural district hospitals. The sustainability of this model depends on the surgical and anaesthetic skills of future generations of GPs. Urban over-loaded regional hospitals may require different public-private models that include out-sourcing rather than in-sourcing of private providers.

**Limitations**

We collected obstetric data from registers and did not perform individual patient folder reviews. The proportion of caesarean delivery complications could have been higher if individual folders were reviewed. Post caesarean sepsis for example could not be identified with the study methodology. Where no indication was listed in the theatre register for a caesarean delivery, this information would have been found had individual folders been reviewed. Furthermore, although a low proportion of women were referred following caesarean delivery, this group could have experienced morbidity or mortality but were not followed up to determine their outcomes at the regional referral hospital.

**Conclusion**

On a broader level, the findings of this study suggest that private GPs can play an important role in filling "gaps" and expanding quality care in the public sector in areas where the total number of doctors or skills and expertise are in short supply, such as in rural areas. In addition, provided that the broader systems and institutional framework to ensure compliance with clinical guidelines and protocols are in place, this can be done in a safe and effective manner; and could be seen as a prototype for NHI in such settings. It is important to acknowledge that this study only explored contracting of private GPs. In the private sector in South Africa deliveries are mostly undertaken by specialist obstetricians and not based on a midwifery model of care.  Therefore, contracting by public hospitals of specialist obstetricians may lead to different outcomes than we observed especially in caesarean delivery rates. Given the high caesarean delivery rate amongst private specialist obstetricians[6] , with rates far higher than global averages or recommended norms, there is a need to review the service delivery model for obstetric care in the private sector prior to extending public-private contracting to specialist obstetricians, in order that public and private sectors can work together without reproducing these inappropriately high caesarean delivery rates. This type of strategic planning is important, to enable effective

For peer review only

implementation of the NHI which aims to harness private resources to serve the needs of the broader South African population.

**Figure legends**

Figure 1: Private GP utilisation and caesarean delivery rate

Figure 2: Utilisation of private GPs and caesarean section profile and outcomes

**Acknowledgements** We thank all the health professionals who took time to participate in our interviews and to the hospital managers for facilitating access to the obstetric registers. We also thank Vishal Brijlal for his comments on a draft of this manuscript.

**Contributors** TD, GS, ED and SF conceptualised the study; TD, GS, ED and YB analysed the data; all authors contributed to the interpretation of the data, writing the paper and reviewing the content.

**Funding** The study was funded by the Bill and Melinda Gates Foundation (Grant Agreement ID INV-023276)

**Competing interests** None declared.

**Patient consent for publication** Not applicable.

**Ethics approval** Ethics approval was granted by the South African Medical Research Council Human Research Ethics Committee (approval number EC003-2/2021) and by the Western Cape Provincial Department of Health and Wellness (approval number WC_202103_017).

**Data availability** The aggregated routine data are available on request to the corresponding author.

For peer review only

## References

1. Trends in maternal mortality 2000-2017. Geneva: World Health Organisation, 2019.
2. National Department of Health. Saving Mothers 2017-2019. Seventh report on confidential enquiries into maternal deaths in South Africa. Pretoria: National Department of Health, 2020.
3. Sobhy S, Arroyo-Manzano D, Murugesu N, et al. Maternal and perinatal mortality and complications associated with caesarean section in low-income and middle-income countries: a systematic review and meta-analysis. *The Lancet* 2019;393(10184):1973-82. doi: 10.1016/S0140-6736(18)32386-9
4. Barber SL, Kumar A, Roubal T, et al. Harnessing the private health sector by using prices as a policy instrument: Lessons learned from South Africa. *Health Policy* 2018;122(5):558-64. doi: 10.1016/j.healthpol.2018.03.018 [published Online First: 2018/04/07]
5. National Department of Health. National Health Insurance Bill. National Department of Health. Pretoria, 2019.
6. Solanki G, Fawcus S, Daviaud E. A cross sectional analytic study of modes of delivery and caesarean section rates in a private health insured South African population. *PloS one* 2019;14(6):e0219020. doi: 10.1371/journal.pone.0219020
7. Amu H, Dickson KS, Kumi-Kyereme A, et al. Understanding variations in health insurance coverage in Ghana, Kenya, Nigeria, and Tanzania: Evidence from demographic and health surveys. *PloS one* 2018;13(8):e0201833. doi: 10.1371/journal.pone.0201833
8. Iyer V, Sidney K, Mehta R, et al. Availability and provision of emergency obstetric care under a public–private partnership in three districts of Gujarat, India: lessons for Universal Health Coverage. *BMJ Global Health* 2016;1(1):e000019. doi: 10.1136/bmjgh-2015-000019
9. Chaturvedi S, Randive B. Public private partnerships for emergency obstetric care: Lessons from Maharashtra. *Indian Journal of Community Medicine* 2011;36(1):21-26. doi: 10.4103/0970-0218.80788
10. Department of Health. A District Hospital Service Package for South Africa. Pretoria: Department of Health, 2002.
11. Gale NK, Heath G, Cameron E, et al. Using the framework method for the analysis of qualitative data in multi-disciplinary health research. *BMC Med Res Methodol* 2013;13:117. doi: 10.1186/1471-2288-13-117
12. Solanki GC, Cornell JE, Daviaud E, et al. Caesarean section rates in South Africa: A case study of the health systems challenges for the proposed National Health Insurance. *SAMJ* 2020;110(8)
13. World Health Organisation. WHO statement on caesarean section rates. Geneva: WHO, 2015.
14. Salazar M, Vora K, Sidney Annerstedt K, et al. Caesarean sections in the in the context of the Chiranjeevi Yojana public private partnership program to promote institutional birth in Gujarat, India; does the embedded disincentive for caesarean section work? *International journal for equity in health* 2019;18(1):17-17. doi: 10.1186/s12939-019-0922-5
15. Borrescio-Higa F, Valdés N. Publicly insured caesarean sections in private hospitals: a repeated cross-sectional analysis in Chile. *BMJ open* 2019;9(4):e024241-e41. doi: 10.1136/bmjopen-2018-024241
16. Fawcus S, Pattinson RC, Moodley J, et al. Maternal deaths from bleeding associated with caesarean delivery: A national emergency. *SAMJ* 2016;106(5)
17. Pattinson RC. Safety versus accessibility in maternal and perinatal care. *SAMJ* 2015;105(4)
18. Randive B, Chaturvedi S, Mistry N. Contracting in specialists for emergency obstetric care- does it work in rural India? *BMC Health Services Research* 2012;12(1):485. doi: 10.1186/1472-6963-12-485

For peer review only

19. Wilson A, Lissauer D, Thangaratinam S, et al. A comparison of clinical officers with medical doctors on outcomes of caesarean section in the developing world: meta-analysis of controlled studies. *Bmj* 2011;342:d2600. doi: 10.1136/bmj.d2600 [published Online First: 2011/05/17]

For peer review only

Figure 1: Private GP utilisation and caesarean delivery rate

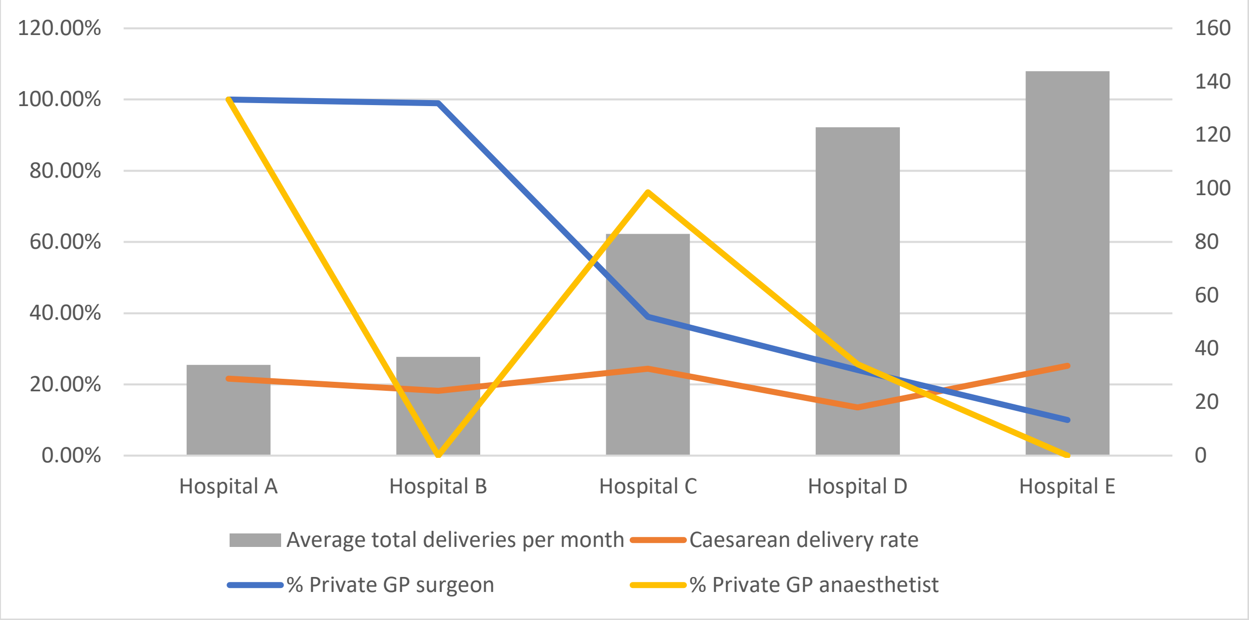

Figure 2: Utilisation of private GPs and caesarean section profile and outcomes

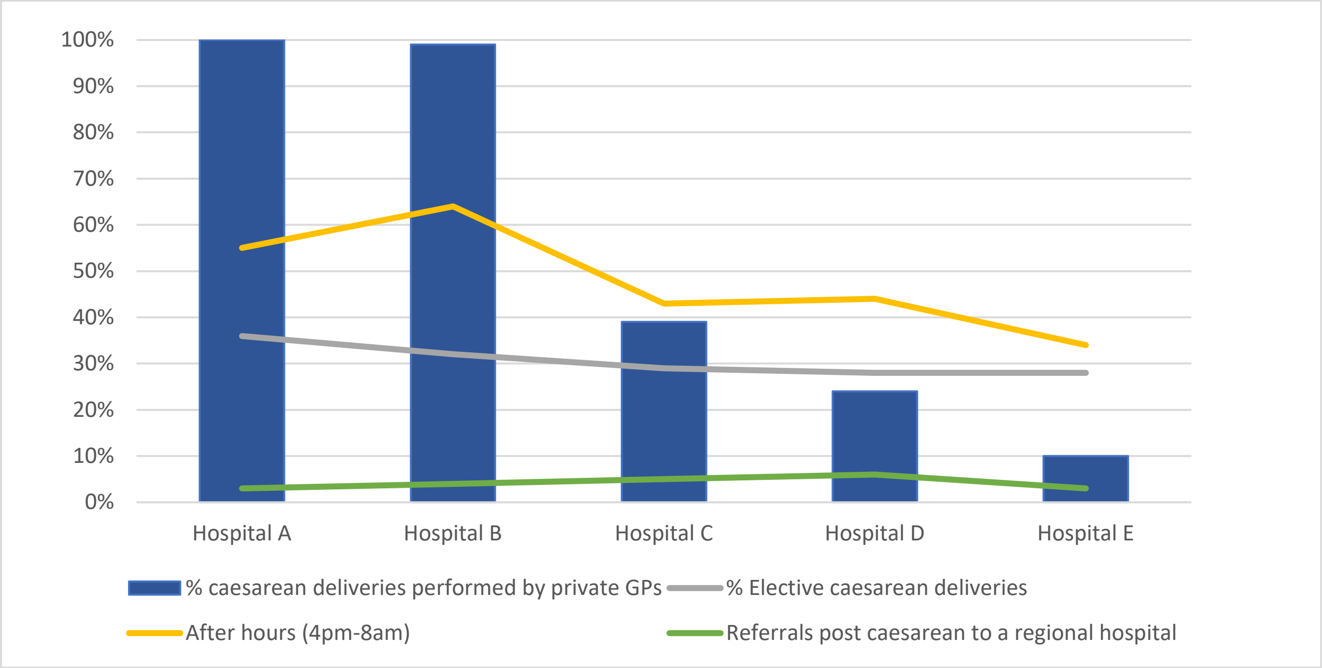

Supplementary tables

Supplementary table 1: Indications for emergency caesarean sections

| Indications | Citrusdal | | Clanwilliam | | Vredendal | | Swartland | | Vredenburg | | All Hospitals | |
|---|---|---|---|---|---|---|---|---|---|---|---|---|
| | N | % | N | % | N | % | N | % | N | % | N | % |
| Fetal distress/abnormal CTG | 15 | 26 | 28 | 51 | 77 | 45 | 79 | 55 | 155 | 49 | 354 | 48 |
| Prolonged labour/CPD | 6 | 10 | 10 | 18 | 24 | 14 | 18 | 12 | 37 | 12 | 95 | 13 |
| Previous C/S (in labour) | 4 | 7 | 3 | 5 | 42 | 25 | 9 | 6 | 12 | 4 | 70 | 9 |
| Malpresentation (obstructed labour) | 12 | 21 | 6 | 11 | 11 | 6 | 11 | 8 | 30 | 10 | 70 | 9 |
| Failed induction | 2 | 4 | 1 | 2 | 2 | 1 | 8 | 6 | 27 | 8 | 40 | 5 |
| placental abruption | 2 | 4 | 1 | 2 | 5 | 3 | 6 | 4 | 3 | 1 | 17 | 2 |
| Pre-eclampsia/ HPT | 0 | 0 | 1 | 2 | 0 | 0 | 4 | 3 | 9 | 3 | 14 | 2 |
| PROM | 3 | 5 | 0 | 0 | 0 | 0 | 0 | 0 | 3 | 1 | 6 | 1 |
| Multiple pregnancy | 1 | 2 | 2 | 4 | 0 | 0 | 2 | 1 | 0 | 0 | 5 | 1 |
| APH (antepartum haemorrhage) | 0 | 0 | 0 | 0 | 0 | 0 | 1 | 1 | 2 | 1 | 3 | 0.4 |
| Failed VBAC | 0 | 0 | 0 | 0 | 0 | 0 | 0 | 0 | 0 | 0 | 0 | 0 |
| Big baby | 0 | 0 | 0 | 0 | 0 | 0 | 0 | 0 | 0 | 0 | 0 | 0 |
| Other | 2 | 4 | 3 | 5 | 4 | 2 | 4 | 3 | 17 | 5 | 30 | 4 |
| Number without listed indication | 10 | 17 | 0 | 0 | 6 | 4 | 1 | 1 | 20 | 6 | 37 | 5 |
| **Grand Total** | **57** | **100.00%** | **55** | **100.00%** | **171** | **100.00%** | **143** | **100.00%** | **315** | **100.00%** | **741** | **100.00%** |

For peer review only

Supplementary table 2: indications for elective caesarean sections

| Indications | Citrusdal | | Clanwilliam | | Vredendal | | Swartland | | Vredenburg | | All Hospitals | |
|---|---|---|---|---|---|---|---|---|---|---|---|---|
| | N | % | N | % | N | % | N | % | N | % | N | % |
| Previous C/S | 5 | 16 | 16 | 61 | 50 | 70 | 40 | 71 | 66 | 55 | 177 | 58 |
| Malpresentation (breech/transverse) | 2 | 6 | 0 | 0 | 4 | 6 | 5 | 9 | 13 | 11 | 24 | 8 |
| HIV & unsuppressed VL | 1 | 3 | 2 | 8 | 0 | 0 | 0 | 0 | 8 | 7 | 11 | 3 |
| Multiple pregnancy | 0 | 0 | 0 | 0 | 1 | 1.4 | 1 | 2 | 3 | 2 | 5 | 2 |
| Big baby | 0 | 0 | 0 | 0 | 1 | 1.4 | 0 | 0 | 3 | 2 | 4 | 1 |
| Patient request | 0 | 0 | 0 | 0 | 0 | 0 | 0 | 0 | 2 | 2 | 2 | 1 |
| Diabetes & macrosomia | 0 | 0 | 0 | 0 | 0 | 0 | 0 | 0 | 1 | 1 | 1 | 0.3 |
| Obstructive vulvar warts | 0 | 0 | 0 | 0 | 0 | 0 | 0 | 0 | 0 | 0 | 0 | 0 |
| Other (genital herpes etc) | 1 | 3 | 1 | 4 | 7 | 10 | 1 | 2 | 10 | 8 | 20 | 7 |
| Number without listed indication | 23 | 72 | 7 | 27 | 8 | 11 | 9 | 16 | 15 | 12 | 62 | 20 |
| **Grand Total** | **32** | **100.00%** | **26** | **100.00%** | **71** | **100.00%** | **56** | **100.00%** | **121** | **100.00%** | **306** | **100.00%** |

For peer review only

BMJ Open

# Utilisation of private general practitioners to provide caesarean deliveries in five rural district public hospitals in South Africa: a mixed-method study

| | |
|---|---|
| Journal: | *BMJ Open* |
| Manuscript ID | bmjopen-2022-067663.R1 |
| Article Type: | Original research |
| Date Submitted by the Author: | 08-Dec-2022 |
| Complete List of Authors: | Doherty, Tanya; South African Medical Research Council, Health Systems Research Unit<br>Solanki, Geetesh; South African Medical Research Council, HSRU<br>Daviaud, Emmanuelle; South African Medical Research Council<br>Bartmann, Yvandi; South African Medical Research Council<br>Hawkridge, Anthony; Western Cape Department of Health<br>Fawcus, Sue ; University of Cape Town Faculty of Health Sciences |
| <b>Primary Subject Heading</b>: | Obstetrics and gynaecology |
| Secondary Subject Heading: | Health services research |
| Keywords: | OBSTETRICS, PUBLIC HEALTH, Organisation of health services < HEALTH SERVICES ADMINISTRATION & MANAGEMENT |
| | |

SCHOLARONE™ Manuscripts

*I, the Submitting Author has the right to grant and does grant on behalf of all authors of the Work (as defined in the below author licence), an exclusive licence and/or a non-exclusive licence for contributions from authors who are: i) UK Crown employees; ii) where BMJ has agreed a CC-BY licence shall apply, and/or iii) in accordance with the terms applicable for US Federal Government officers or employees acting as part of their official duties; on a worldwide, perpetual, irrevocable, royalty-free basis to BMJ Publishing Group Ltd ("BMJ") its licensees and where the relevant Journal is co-owned by BMJ to the co-owners of the Journal, to publish the Work in this journal and any other BMJ products and to exploit all rights, as set out in our licence.*

*The Submitting Author accepts and understands that any supply made under these terms is made by BMJ to the Submitting Author unless you are acting as an employee on behalf of your employer or a postgraduate student of an affiliated institution which is paying any applicable article publishing charge ("APC") for Open Access articles. Where the Submitting Author wishes to make the Work available on an Open Access basis (and intends to pay the relevant APC), the terms of reuse of such Open Access shall be governed by a Creative Commons licence – details of these licences and which Creative Commons licence will apply to this Work are set out in our licence referred to above.*

*Other than as permitted in any relevant BMJ Author's Self Archiving Policies, I confirm this Work has not been accepted for publication elsewhere, is not being considered for publication elsewhere and does not duplicate material already published. I confirm all authors consent to publication of this Work and authorise the granting of this licence.*

For peer review only

# Utilisation of private general practitioners to provide caesarean deliveries in five rural district public hospitals in South Africa: a mixed-method study

Authors

Tanya Doherty[1,2], Geetesh Solanki[1], Emmanuelle Daviaud[1], Yvandi Bartmann[1], Anthony Hawkridge[3], Sue Fawcus[4]

Affiliations

[1] Health Systems Research Unit, South African Medical Research Council, Cape Town, South Africa

[2] Department of Paediatrics and Child Health, University of Cape Town, Cape Town, South Africa

[3] Swartland Subdistrict, Western Cape Department of Health, Malmesbury, South Africa

[4] Department of Obstetrics and Gynaecology, University of Cape Town, Cape Town, South Africa

**Correspondence to** Prof Tanya Doherty tanya.doherty@mrc.ac.za



For peer review only - http://bmjopen.bmj.com/site/about/guidelines.xhtml

Abstract

Objective

Researching how public-private engagements may promote universal access to safe obstetric care including caesarean delivery is essential. The aim of this research was to document the utilisation of private general practitioners (GPs) contracted to provide caesarean delivery services in five rural district hospitals in the Western Cape, the profile and outcomes of caesarean deliveries. We also describe stakeholder experiences of these arrangements in order to inform potential models of public private contracting for obstetric services.

Methods

We utilised a mixed-method study design to describe rural district hospitals' utilization of private GP contracting for caesarean deliveries. Between April 2021 and March 2022 we collated routine data from delivery and theatre registers to capture the profile of deliveries and maternal outcomes. We conducted 23 semi-structured qualitative interviews with district managers, hospital employed doctors and private GPs to explore their experiences of the contracting arrangements.

Results

The use of private GPs as surgeon or anaesthetist for caesarean deliveries differed widely across the hospitals. Overall, the utilisation of private GPs for anaesthetics was similar (29% of all caesarean deliveries) to the utilisation of private GPs as surgeons (33% of all caesarean deliveries). The proportion of caesarean deliveries undertaken by private GPs as the primary surgeon was inversely related to size of hospital and mean monthly deliveries. Adverse outcomes following a caesarean delivery were rare. Qualitative data provided insights into contributions made by private GPs and the contracting models which did not incentivize over-servicing.

Conclusion

The findings of this study suggest that private GPs can play an important role in filling gaps and expanding quality care in rural public facilities that have insufficient obstetric skills and expertise. Different approaches to enable access to safe caesarean delivery are needed for different contexts and contracting with experienced private is one resource for rural district hospitals to consider.




For peer review only - http://bmjopen.bmj.com/site/about/guidelines.xhtml

**Strengths and Limitations**

- This is one of the first studies to describe utilisation of private GPs to support caesarean deliveries within rural district hospital settings in a low-and-middle income country context.
- A mixed-method study design including routine obstetric data and qualitative interviews provides a robust description of experiences with this public-private model.
- We collated obstetric data from registers and did not perform individual patient folder reviews. The proportion of caesarean delivery complications could have been higher if individual folders were reviewed.
- We did not collect data on neonatal outcomes which is also an important indicator of the quality and safety of caesarean delivery.

For peer review only

## Introduction

Maternal and child health is a major public health concern in South Africa (SA). Whilst there has been progress in reducing maternal mortality to 119 per 100 000 live births,[1] there remain huge disparities between public and private sectors in the distribution of obstetric care providers, models of care and outcomes.[2] The caesarean delivery rate in the public sector is 28%[2] compared to 74% in the private sector.[3] A global analysis exploring the relationship between health financing indicators and caesarean delivery rates found that in 2018 there were a total of 8.8 million unnecessary caesarean deliveries globally, two-thirds of which occurred in upper middle-income countries.[4] The study reported that a 10 per cent increase in voluntary health insurance was associated with a 4 per cent increase in excess caesarean deliveries.[4]

In line with patterns seen in other low-and-middle-income countries,[5] the case fatality rate (CFR) for caesarean deliveries in the SA public sector is three times higher than for vaginal delivery and 27% of the caesarean delivery related CFR is associated with haemorrhage.[2] Caesarean delivery CFR and hemorrhage rates are not routinely measured in the private sector so such data is not available. The 2014-2016 Saving Mothers report for SA described a review of maternal deaths in the private sector. The maternal mortality rate was 45 per 100,000 live births (one third that of the national SA rate at the time) with a similar proportion of deaths from obstetric hemorrhage (19%) of which 17% were from caesarean delivery associated bleeding.[6] Improved clinical governance and skills development in the public sector are critical to reducing this disparity, but these efforts could be complemented by the engagement of health professionals with obstetric skills in the private sector.

In efforts towards achieving Universal Health Coverage (UHC), SA is in the process of developing policy to allow implementation of a National Health Insurance (NHI). The NHI Bill[7] proposes that NHI will be a strategic purchaser of health care services for the entire SA population. The proposed NHI will contract for obstetric services from both public and private providers. A key challenge in SA regardless of when and in what form NHI is implemented, is to develop contracting models that allow public and private resources to be mobilized to the benefit of the entire population. This is particularly important for rural district hospitals where surgical skills and supervision may be insufficient to meet the service requirements for obstetric care, and specialist services are not readily available in the public or private sectors.



For peer review only - http://bmjopen.bmj.com/site/about/guidelines.xhtml

However, attempts to harness the resources of the private sector to service the needs of the broader SA population would need to address the challenge of ensuring that the care provided is appropriate and patterns of inappropriate care, including the high caesarean delivery rate of the private sector[3], is not reproduced for the entire population. The development of contracting models that would best mitigate these risks is critical in delivering UHC.

Researching and documenting how private sector resources can be harnessed to support the public health system is urgently needed now, in the transition, and full implementation phases of NHI in SA and also to inform developments in other African countries that are implementing similar national health insurance financing arrangements. These include countries such as Ghana, Kenya, Nigeria and Tanzania.[8] There are some lessons to be learnt from public-private contracting for obstetric services in India where states have implemented different models of contracting arrangements to increase access to obstetric care[9]. In some states private obstetricians are contracted in to public facilities to increase skills and human resources while in other states costs for services are subsidized within private facilities.[10]

Research to understand whether public-private engagements could reduce inequities and promote universal access to safe obstetric care including caesarean delivery is essential. Understanding the nature of public-private engagements in the area of obstetric care is particularly important because caesarean delivery is the most commonly performed surgical procedure and the findings may also be useful in informing strategies for other medical and surgical disciplines where similar patterns of under servicing and over-servicing in the public vs. private sector may occur.

We carried out research to document the utilisation of private general practitioners (GPs) contracted to provide caesarean delivery services in five rural district hospitals in the Western Cape and to qualitatively describe the experiences and perspectives of managers and doctors involved with a view to informing the development of public private arrangements for improving obstetric and maternal outcomes in rural areas in South Africa and LMICs more broadly. The aim of this paper is to document our findings on the utilisation of the GP's, the profile and outcomes of caesarean deliveries. We also describe stakeholder experiences of these arrangements in order to inform the development of models of public private contracting for obstetric services.



For peer review only - http://bmjopen.bmj.com/site/about/guidelines.xhtml

## Methods

### Study design

We undertook descriptive health systems research utilising a mixed-method study design to describe rural district hospitals' utilization of private GP contracting for caesarean deliveries in the Western Cape province and the profile and maternal outcomes of these deliveries in five hospitals. We chose a mixed-method study design incorporating both quantitative and qualitative data collection as this approach is well suited to applied health systems research.[11 12]

### Study setting

The setting for this research was the Western Cape province where existing public-private contracting for caesarean delivery services was occurring due to human resource shortages in rural district hospitals. Five rural district hospitals within one rural district were chosen following engagement with provincial managers and obstetric clinical managers.

In South Africa, women with low-risk pregnancies receive antenatal care at primary care clinics and community health centres. District hospitals provide level 1 (generalist) services to in-patients and outpatients including obstetric care for women with low-risk pregnancies. District hospitals have between 30 and 200 beds, a 24-hour emergency service and an operating theatre. Generalists (medical officers) provide the services together with nursing staff and allied health professionals, some district hospitals have specialist family physicians serving as clinical managers but there are no obstetric or anaesthetic specialists at district hospital level. Most district hospitals also have community service doctors. These are doctors who have completed a two-year internship and are required to complete a further one year of community service.[13] None of the five hospitals had newly qualified intern medical doctors who are generally not placed within district hospitals.

For obstetric services at district hospital level normal vaginal deliveries are performed by midwives, assisted vaginal deliveries are performed by advanced midwives or medical officers and caesarean deliveries (surgery and anaesthesia) are performed by medical officers. Pregnant women with pre-existing morbidities such as diabetes, autoimmune disorders, thyroid disease, and cardiac disease or obstetric complications such as anticipated preterm delivery, suspected intra-uterine growth restrictions, pre-eclampsia, placenta praevia, abruptio placentae, multiple pregnancy, two previous



For peer review only - http://bmjopen.bmj.com/site/about/guidelines.xhtml

caesarean sections, Body Mass Index over 35-40 kgs/m², and severe anaemia are referred for delivery to a secondary or tertiary level hospital.

Public health facilities are permitted to contract the services of private providers where needed. There are three mechanisms by which private providers can be contracted to the public service: through a locum agency, through a sessional contract which is limited to a maximum of 39 hours per month or as a service provider in response to a tender for specific services. In all three contracting models the remuneration is time-based and not related to the number of patients or theatre cases performed. In the case of obstetric services private providers are mainly utilized for theatre services either as a GP surgeon or GP anaesthetist to undertake caesarean deliveries or for obstetric surgery including ectopic pregnancy, termination of pregnancy and dilatation and curettage following spontaneous miscarriage. They may also be called for an assisted delivery if the establishment doctor is unable to manage a complicated delivery. For GPs contracted through a sessional contract, medico-legal indemnity is provided by the state but for those contracted as locums or through a service provider tender they are required to have their own medico-legal indemnity cover. In these five hospitals the private GPs did not have medical indemnity for private obstetric practice and only performed caesarean deliveries during their public sector contracted time.

Quantitative data collection

For a period of 12 months (1 April 2021 – 31 March 2022) we collated quantitative obstetric clinical data from all five participating hospitals. Clinical outcomes were captured from the delivery register, theatre register and obstetric transfer book. Outcomes that could be collated from these sources without individual patient folder review included: mode of delivery and caesarean delivery complications (maternal death, postpartum haemorrhage (PPH) and referral to a regional hospital). Other procedures performed at or after a caesarean delivery were also captured as a measure of complications (hysterectomy, B-lynch suture and re-look laparotomy). For each caesarean delivery we captured whether the surgeon and anaesthetist was a hospital employed medical officer or a private GP, the type of anaesthetic given, whether the procedure was classified as an emergency or elective and whether it was performed during daytime or evening/night time (4pm – 8am, Monday- Sunday).



For peer review only - http://bmjopen.bmj.com/site/about/guidelines.xhtml

Clinical data was collated from registers by research nurses recruited for the purpose of the study. The monthly data was entered into a pre-set excel spreadsheet.

Qualitative data collection

Semi-structured interview guides were developed, one for private providers and one for public providers. Qualitative interviews were undertaken by three of the investigators (TD, GS and ED) at the hospitals and district office. The investigators who conducted interviews are all senior researchers with experience in qualitative interviewing. Two were health economists and one a health systems researcher. They had no prior relationships with any of the interviewees. All interviews were conducted in a private consulting room or office and were undertaken in English.

Interviews were undertaken with private GPs who had entered into public service contracts with each of the five hospitals to explore their perceptions of undertaking work in the public sector and the role they played. At each hospital we also interviewed the hospital managers and public providers (doctors) to explore their perceptions of the public/ private interface including clinical decision making, private GP availability and remuneration models. Types of questions asked to private GPs included: ‘’ What has been your experience of working in government hospitals? and ‘How do you balance your time between private practice and government hospital work?’’; types of questions asked to district managers and government doctors included: ‘’ What services are private GPs contracted to provide?, ‘’How are decisions about caesarean delivery made within the clinical team? , ‘’How are private GPs remunerated for their time by the public sector?’’ During interviews we also collected information on the total number of hours per month each GP was contracted to the public hospital and used this information to calculate the number of fulltime equivalent doctor posts the GPs contributed at each hospital. For example, in hospital C there were 5 private GPs each contracted for 25 hours per week. A public sector doctor works 56 hours per week therefore in this hospital the combined private GP hours are equivalent to 2.2 fulltime equivalent establishment doctors.

We undertook a total of 23 semi-structured qualitative interviews; including 3 district managers (2 male, 1 female), twelve private GPs (7 male, 5 female) and 8 government employed doctors (4 male, 4 female).



For peer review only - http://bmjopen.bmj.com/site/about/guidelines.xhtml

## Quantitative data analysis

Quantitative data from hospital records was analysed in excel using simple descriptive mean and standard deviation (mean monthly number of deliveries) and proportions (caesarean delivery rate, deliveries undertaken by a private GP, elective caesarean deliveries and caesarean deliveries occurring during daytime or evening/ night time hours) stratifying by hospital over the 12 month data collation period.

We describe the proportion of total annual caesarean deliveries at each hospital undertaken by private GPs as surgeon or anaesthetist and explore the relationship between type of provider and the caesarean delivery profile (elective vs emergency and timing in terms of day time hours or during the evening/night) and adverse outcomes (referral to a regional hospital following a caesarean delivery, maternal death, postpartum haemorrhage and other procedures performed at caesarean deliveries).

## Qualitative data analysis

Qualitative interviews were digitally recorded and transcribed. A framework analysis[14] approach was applied which is well suited to implementation research designs. We drew on a framework previously developed by members of our research team which outlines drivers, challenges and required action for obstetric care in preparing for NHI.[15] The interviews were read and re-read by members of the team to familiarise ourselves with the content. Emerging categories and major themes were identified and mapped against the framework and quantitative findings. Five members of the research team (TD, ED, GS, SF and YB) read transcripts and met to discuss emerging categories and major themes. Four main themes were identified which included: the role played by private GPs contracted to district hospitals; the nature of clinical decision making regarding caesarean delivery; balancing work between private practice and public sector and perspectives on remuneration models. The qualitative data enhance contextual understanding of the quantitative outcomes related to CS rates and outcomes by shedding light on provider experiences and perceptions of aspects such as workload and remuneration.

## Ethical considerations

Ethics approval was granted by the South African Medical Research Council Human Research Ethics Committee (approval number EC003-2/2021) and permission was also granted by the Western Cape Provincial Department of Health and Wellness (approval number WC_202103_017). No personal identifying information was captured from hospital records, only aggregated summary information.



For peer review only - http://bmjopen.bmj.com/site/about/guidelines.xhtml

All health professionals who were approached to participate in an interview were read an information sheet outlining the expectations in terms of length of the discussion, the voluntary nature of the participation and measures to ensure confidentiality. Health professionals who agreed to participate signed an informed consent form.

**Patient and public involvement** Patients and/or the public were not involved in the design, or conduct, of this research. We will be disseminating the findings including through presentations at each of the participating hospitals which will be targeted at stakeholder groups including patients and members of the public.

## Results

### Hospital characteristics and utilisation of private GPs for caesarean deliveries

The five district hospitals ranged in size from 35 beds in hospital A to 118 beds in hospital E. Over the period 1 April 2021 to 31 March 2022, the average number of monthly deliveries ranged from 34 in hospital A to 144 in hospital E. None of the hospitals has primary care clinics in its catchment areas that perform vaginal deliveries other than in the case of emergencies and only during daytime hours. All five hospitals have some involvement of private GPs to assist with the obstetric service but the level of utilisation of their services differed greatly across the hospitals. In hospital A there are no government employed doctors and the hospital is run entirely by a private GP practice consisting of 5 doctors who provide 24-hour cover to the hospital. Across the other hospitals the number of contracted GPs ranged from 1 in hospital E to 5 in hospital C but this translated into a maximum of 2 full time equivalent doctors in hospital C and 0.2 in hospital E (table 1).



For peer review only - http://bmjopen.bmj.com/site/about/guidelines.xhtml

Table 1: Characteristics of participating hospitals

| Hospital | Hospital A | Hospital B | Hospital C | Hospital D | Hospital E |
|---|---|---|---|---|---|
| Number of beds | 35 | 50 | 75 | 85 | 118 |
| Number of maternity beds (antenatal, labour and postnatal) | 8 | 10 | 12 | 14 | 25 |
| Number of clinics in the hospital catchment that perform vaginal deliveries | 0 | 5 clinics that can do emergency vaginal deliveries if required, no 24 hour service | 0 | 1 community day clinic, 4 clinics and 6 satellite services, none offer 24 hour service, can do emergency vaginal deliveries if required | 10 clinics that can do emergency vaginal deliveries if required, no 24 hour service |
| Mean monthly number of deliveries (SD) | 34 (7.2) | 37 (9.7) | 83 (5.2) | 123 (9.8) | 144 (18.3) |
| Number of fulltime hospital employed doctors | 0 | 6 | 6 | 10 (2 of which are specialist family physicians) | 13 |
| Number of community service doctors included in the total medical staff | 0 | 4 | 2 | 4 | 5 |
| Number of privately contracted GPs to assist with the obstetric service | One practice consisting of 5 GPs providing a 24- hour service undertaking both surgery and anaesthesia | 2 GPs undertaking surgery | 5 (2 for anaesthetics and 3 for surgery) | 4 (2 surgical, 2 anaesthetic); 1 GP has a post-graduate diploma in anaesthetics, 1 GP has a post-graduate diploma in obstetrics, 1 GP is a specialist family physician | 1 GP doing surgery only |
| Number of full-time equivalent private GPs | 0.9 | 0.3 | 2.2 | 0.9 | 0.2 |

SD= standard deviation



For peer review only - http://bmjopen.bmj.com/site/about/guidelines.xhtml

Table 2 shows the profile of deliveries occurring in the hospitals over the 12-month study period. The proportion of normal vaginal deliveries ranged from 74% in hospitals C and E to 85% in hospital D. Assisted vaginal deliveries were uncommon (<3%) across all five hospitals. The caesarean delivery rate ranged from 14% in hospital D to 25% in hospital E. Spinal anaesthesia was used in 95% of all cases.

The use of private GPs as surgeon or anaesthetist for caesarean deliveries differed widely across the five hospitals. Overall, the utilisation of private GPs for anaesthetics was similar to (29% of all caesarean deliveries) the utilisation of private GPs as surgeons (33% of all caesarean deliveries) although in hospital C more private GPs were utilised for anaesthetics than for surgery (Table 2). Interviews with both private and hospital employed doctors revealed that the skills shortage was perceived to be surgical rather than anaesthetic, and that private GPs make a contribution to training community service doctors in surgical skills for caesarean deliveries as these doctors explained:

*I'm doing more the cutting side. So, I mean, the comserves (community service doctor) can't cut. I am always cutting. There's one comserve on and it's me. So, we cut with the sister (Private GP, Hospital B).*

*So, in a month I must train all the community service doctors to be safe to do caesarean deliveries and ectopics which is not an easy undertaking (Clinical manager, Hospital D).*

*That is just the GP that is usually the primary surgeon. I am more of the assistant. I'm still learning to do caesars on my own. So, it's usually just the GPs who are doing the primary surgery. On the anaesthetic side there's another GP that is on call. We're basically an assistant. I'm still learning so I mostly doing primi-gravida's at the moment (Year 1 medical officer, Hospital C).*

*I'm on call every second day of my life and every second weekend of my life because we, as the two of us (private GPs), are the only people that can do caesarean deliveries. The rest of the people are either young MOs with little experience or, community service doctors that cannot do that (Private GP, Hospital B).*

For peer review only



For peer review only - http://bmjopen.bmj.com/site/about/guidelines.xhtml

Table 2: Summary of deliveries and private GP utilisation 01 April 2021 – 31 March 2022

| | Hospital A | | Hospital B | | Hospital C | | Hospital D | | Hospital E | | All Hospitals | |
|---|---|---|---|---|---|---|---|---|---|---|---|---|
| **Delivery profile** | **N** | **%** | **N** | **%** | **N** | **%** | **N** | **%** | **N** | **%** | **N** | **%** |
| Normal vaginal delivery (NVD) | 311 | 75 | 356 | 80 | 732 | 74 | 1,259 | 85 | 1,271 | 74 | 3,929 | 78 |
| Assisted vaginal (vacuum or forceps) | 12 | 3 | 9 | 2 | 17 | 2 | 13 | 1 | 21 | 1 | 72 | 1 |
| Caesarean deliveries | 89 | 22 | 81 | 18 | 242 | 24 | 199 | 14 | 436 | 25 | 1,047 | 21 |
| **Total** | **412** | | **446** | | **991** | | **1,471** | | **1,728** | | **5,048** | |
| **Private GP utilisation for caesarean deliveries** | | | | | | | | | | | | |
| **Surgeon** | | | | | | | | | | | | |
| Public Sector non specialist Surgeon | 0 | 0 | 1 | 1 | 148 | 61 | 152 | 76 | 394 | 90 | 706 | 67 |
| Private GP Surgeon | 89 | 100 | 80 | 99 | 94 | 39 | 47 | 24 | 42 | 10 | 341 | 33 |
| **Anaesthetist** | | | | | | | | | | | | |
| Public sector non specialist anaesthetist | 0 | 0 | 81 | 100 | 63 | 26 | 148 | 74 | 436 | 100 | 746 | 71 |
| Private GP Anaesthetist | 89 | 100 | 0 | 0 | 179 | 74 | 51 | 26 | 0 | 0 | 300 | 29 |
| **Profile of caesarean deliveries** | | | | | | | | | | | | |
| **Hours, Monday-Sunday:** | | | | | | | | | | | | |
| Evening/ night-time (4pm-8am) | 49 | 55 | 52 | 64 | 103 | 43 | 88 | 44 | 150 | 34 | 442 | 42 |
| Daytime hours (8am-4pm) | 40 | 45 | 29 | 36 | 139 | 57 | 111 | 56 | 286 | 66 | 605 | 58 |
| **Elective vs. Emergency** | | | | | | | | | | | | |
| Elective caesarean | 32 | 36 | 26 | 32 | 71 | 29 | 56 | 28 | 121 | 28 | 306 | 29 |
| Emergency caesarean | 57 | 64 | 55 | 68 | 171 | 71 | 143 | 72 | 316 | 72 | 742 | 71 |
| **Anaesthetic Type** | | | | | | | | | | | | |
| Spinal | 86 | 97 | 79 | 99 | 218 | 92 | 192 | 96 | 414 | 95 | 989 | 95 |
| General anaesthetic | 3 | 3 | 1 | 1 | 16 | 7 | 5 | 3 | 20 | 4.5 | 45 | 4 |
| Combined spinal and general | 0 | 0 | 0 | 0 | 3 | 1 | 2 | 1 | 2 | 0.5 | 7 | 1 |



For peer review only - http://bmjopen.bmj.com/site/about/guidelines.xhtml

The proportion of caesarean deliveries undertaken by private GPs as the primary surgeon was inversely related to size of hospital and total average monthly deliveries (figure 1). The two smallest hospitals were most reliant on private GPs to perform caesarean deliveries and as size of hospital increased fewer caesarean deliveries were undertaken by private GPs. The caesarean delivery rate was not higher in hospitals where private GPs carried out a higher proportion of caesarean procedures (figure 1). During interviews the reasons given for this were that it is generally the hospital-based staff who make the decision about whether a caesarean delivery is required, and the private GP is not involved in that decision. In hospital A, which is run entirely by private GPs the midwives managing the labour ward assess progress of labour and call the GPs from their practice if they assess that a caesarean is required. The following quotes illustrate the decision-making around caesarean deliveries:

*The contract that I have is explicitly for theatre work only. Nothing else. I don't go into labour ward, maternity ward. I get called and I come straight to theatre. The decisions have been made by the medical officers in the ward or in casualty and then I'm the technician. I get called in to do mostly caesars but also ectopic pregnancies and incomplete abortions and that sort of thing. And complications of pregnancy, cervical tears, vaginal tears that sort of thing, retained placentas, but it's only theatre work (Private GP, Hospital E).*

*They book them. We don't really see the patient. We do the surgeries for them just in terms of logistics, and then they follow up with the aftercare after that (Private GP, Hospital C).*

**Profile and outcomes of caesarean deliveries with private GP contracting**

The majority (71%) of caesarean deliveries across all five hospitals were emergency procedures (table 2). The most common indication for an emergency caesarean delivery was foetal distress or an abnormal CTG (48%) (supplementary table 1). Amongst elective caesarean deliveries the main indication was a previous caesarean delivery (58%). For 5% of emergency caesarean deliveries and 20% of elective caesarean deliveries the indication was missing from the theatre register. Individual hospital documentation style differed with a higher proportion of elective caesarean deliveries (72%) with a documented indication missing at hospital A, which was serviced only by private GPs, whilst this was less than 30% at the other four hospitals. However, this did not affect the overall CS rate (supplementary table 2).

Adverse outcomes following a caesarean delivery indicated by blood loss >1000mls, maternal death post caesarean delivery and referral after caesarean delivery to a regional hospital were rare across all



For peer review only - http://bmjopen.bmj.com/site/about/guidelines.xhtml

hospitals with a 1% prevalence of PPH and 4% of women being transferred to a regional hospital following caesarean delivery. Over the 12-month period of data collection there were no emergency procedures (hysterectomy, B-lynch suture or re-look laparotomies) performed during or following a caesarean delivery at any of the hospitals (table 3).

Figure 2 shows the utilisation of private GPs for caesarean deliveries and the profile and outcomes of these deliveries. Slightly over half of all caesarean deliveries were performed during public sector working hours (8am-4pm) but this differed across the hospitals. In hospitals A and B the majority of caesarean deliveries were performed during evening / night time (4pm – 8am). In these two hospitals almost all of the caesarean deliveries are performed by private GPs who consult in their private practices during the day. Their time availability for hospital work is therefore more restricted in day-time hours as these participants explained:

*So, we come to hospital from eight to ten in the morning. Sometimes it does take longer and you only get down there at half past ten. So, from ten to twelve it's like private practice, then clinics, and then from half past two to half past four, five-ish at the practice again* (private GP, Hospital B).

*If it was a GP that was covering, they would be a bit reluctant to come in at four to assist us because having their own practice, they might still have practice till five or maybe six sometimes. So, they would maybe push the hour that they would be available to come in, a little bit further on, but, usually, if we call for a caesar, it's usually a foetal distress, we don't just cut to cut if it's not a foetal distress then we would say we could wait another half an hour (medical officer, hospital E).*

There is no noticeable increase in elective caesarean deliveries with a higher proportion of total caesarean deliveries undertaken by private GPs and rates of referral to a regional hospital following caesarean delivery was low irrespective of the utilisation of private GPs.

The funding models used to remunerate private GPs for their work within the public sector is a rate per shift irrespective of the number or type of procedures performed. Several GPs described the remuneration model during interviews:

*I do one week per month from Monday night, four p.m. till eight a.m. the next Monday and the remuneration is not affected by the number of call outs. It's a flat rate. Whether I get called once in that week or twenty times in that week, the remuneration is the same. Payment every month exactly the same. If I'm lucky, I get called out only once or twice for the week, but, a week ago, I was called out three times in*



For peer review only - http://bmjopen.bmj.com/site/about/guidelines.xhtml

*one night. An ectopic pregnancy, abruptio and, obstructed labour. Just like that. It's four hours in theatre (private GP, Hospital E).*

*You never know how your hours are going to turn out for that week. So, say this week I have a very good week, I only have to be there from eight till eleven. They never phoned me during the night; they never phoned me during my day, the guy who's on next week they're phoning the whole time because it's a caesarean after caesarean. Every night there is an ectopic or a third-degree tear or something so you can't really, you can't really compare the two calls, but everyone still gets the same fixed rate (Private GP, Hospital C).*

*Because the sessional guys (private GPs) are just for the sessions. So, I mean they don't get paid extra if they do ten or one (caesarean deliveries) (Clinical manager, hospital D).*

For peer review only



For peer review only - http://bmjopen.bmj.com/site/about/guidelines.xhtml

Table 3: Adverse outcomes following caesarean deliveries

| | Hospital A | | Hospital B | | Hospital C | | Hospital D | | Hospital E | | All Hospitals | |
|---|---|---|---|---|---|---|---|---|---|---|---|---|
| | N | % | N | % | N | % | N | % | N | % | N | % |
| **Adverse Outcomes** | | | | | | | | | | | | |
| Total PPH/estimated blood loss >=1000 mls | 0 | 0 | 1 | 1 | 2 | 1 | 0 | 0 | 10 | 2 | 13 | 1 |
| Maternal deaths post caesarean | 0 | 0 | 0 | 0 | 1* | 0.4 | 0 | 0 | 0 | 0 | 1 | 0.1 |
| Referrals post caesarean to a regional hospital | 3 | 3 | 3 | 4 | 12 | 5 | 11 | 6 | 11 | 3 | 40 | 4 |
| **Procedures performed at caesarean deliveries** | | | | | | | | | | | | |
| IUCD/tubal ligation | 13 | 15 | 5 | 6 | 33 | 14 | 16 | 8 | 43 | 10 | 110 | 11 |
| Other Procedures | 0 | 0 | 0 | 0 | 0 | 0 | 5 | 3 | o | 0 | 5 | 0.5 |
| Hysterectomies at/after caesarean | 0 | 0 | 0 | 0 | 0 | 0 | 0 | 0 | 0 | 0 | 0 | 0 |
| B lynch suture/balloon tamponade | 0 | 0 | 0 | 0 | 0 | 0 | 0 | 0 | 0 | 0 | 0 | 0 |
| Relook laparotomies | 0 | 0 | 0 | 0 | 0 | 0 | 0 | 0 | 0 | 0 | 0 | 0 |

PPH = postpartum haemorrhage; IUCD = intrauterine contraceptive device * cause of death was eclampsia

For peer review only



For peer review only - http://bmjopen.bmj.com/site/about/guidelines.xhtml

## Discussion

This health systems research study explored the utilisation of private GPs to provide caesarean delivery services in rural district hospitals in the Western Cape. We found that the extent of utilisation of private GPs was inversely related to the size of hospital and number of deliveries. The two larger hospitals with over 100 monthly deliveries had a critical mass of doctors to cover the roster and used private GPs less frequently.

In terms of the delivery profile, we found an overall caesarean delivery rate of 21%. This is higher than the WHO recommended rate of 5-15% of all births[16] but lower than the public sector rate for the Western Cape of 29%.[2] Caesarean delivery rates in district hospitals are lower than the national rate because such hospitals refer women with complications such as two previous caesarean deliveries, pre-eclampsia, placenta praevia, etc. to regional or tertiary hospitals. Also, they deliver low risk women having normal vaginal deliveries because they do not have surrounding 24-hour midwife units, so these low-risk births are included in the denominator. Hospital D's caesarean delivery rate (14%) may be lower because the service was closed for several years following an extensive fire in March 2017, with all caesarean deliveries being done elsewhere. The caesarean delivery service resumed again in February 2021 just prior to the start of data collection for this study. This hospital is also unique in being the only one of the five with specialist family physicians overseeing the clinical services. All caesarean deliveries are discussed 24/7 with a family physician prior to being performed, unless such a discussion may delay safe delivery, which may also contribute to the lower rate of caesarean deliveries.

We did not find an increased caesarean delivery rate in hospitals where a high proportion of caesarean deliveries were performed by private GPs compared to government employed generalist doctors and no increase in caesarean deliveries performed for elective indications. The public-private partnership arrangements in these hospitals are such that the decision-making regarding the need for a caesarean delivery is undertaken by a hospital employee (doctor or midwife) and the private GP is notified once the decision has been made. However, we did find a higher proportion of elective caesarean deliveries without stated indication and a higher proportion performed after 4pm (during evenings and at night) at hospitals more reliant on private GPs. Balancing time between private practice and public sector work may pose a risk to public hospitals where there are delays in private GP availability in emergency situations.



For peer review only - http://bmjopen.bmj.com/site/about/guidelines.xhtml

The remuneration model for GP contracting, with a fixed rate irrespective of number of caesarean deliveries should disincentivize over-servicing. A public-private partnership programme in Gujarat state, India provides private obstetricians with a fixed sum per 100 births to provide care to eligible women below the poverty line. The remuneration model was specifically chosen as a disincentive to prevent unnecessary caesarean deliveries.[17] By comparison in Chile, where women covered under public health insurance can choose to give birth within a selected group of private health facilities the caesarean delivery rate was found to be 71% compared to 26% amongst publicly insured women giving birth in public health facilities. Publicly insured women choosing to give birth in a private facility receive a voucher that covers the costs of the delivery except for a 25% co-payment, the sum of which is dependent on the mode of delivery, which is the responsibility of the patient.[18]

In terms of caesarean delivery outcomes our results suggest that in rural district hospitals utilising experienced private GPs to perform caesarean deliveries there was no trend towards increased adverse outcomes. The rate of complications post caesarean section was low across all five hospitals which is an indication of safe caesarean section deliveries overall in this setting.

There was one maternal death following caesarean delivery in the one-year period of review due to eclampsia, a 1% rate of PPH at caesarean delivery and a very low rate of referral to a regional hospital following caesarean delivery. The rate of PPH at caesarean delivery was low. This is likely to reflect the lower risk caesarean deliveries that were performed at district hospitals, good labour ward management with early recourse to caesarean delivery, and surgical skills. It is possible that some women were referred to higher level hospitals for bleeding after caesarean delivery, but this information is not available because individual folders were not reviewed, and the numbers would be few because overall referral numbers were low.

In SA, district hospitals account for 38% of public sector caesarean deliveries and the case fatality rate for caesarean deliveries at this level of care is 62 per 100,000 caesarean deliveries.[2] Since these hospitals do not have specialist obstetricians or anaesthetists, surgical and anaesthetic skills training and supervision is critical. Deaths due to bleeding at or following a caesarean delivery have been increasing in SA and lack of skilled doctors has been identified as an important avoidable factor.[19] This model of a public-private partnership makes an important contribution to skills development in rural district hospitals through drawing on the skills and experience of private GPs, and in some hospitals private



For peer review only - http://bmjopen.bmj.com/site/about/guidelines.xhtml

family physicians, to perform caesarean deliveries alongside hospital-based medical officers many of whom are undertaking community service or recently qualified.

Balancing access with safety is a critical factor in obstetric care. Ideally facilities that can provide care for uncomplicated births should be close to women's homes and within reasonable distance of a district hospital that can perform caesarean deliveries. For small district hospitals with a low monthly delivery rate, maintaining skills to deal with emergencies is a challenge. To be safe and cost-effective, it has been recommended that district hospitals would need to perform between 500 and 1200 deliveries per year.[20] There should also always be a minimum of two doctors on call (one for anaesthesia and one for surgery), so that caesarean deliveries may be performed on a 24-hours-a-day, 7-days-a-week basis.[20] Hospitals A and B in this study performed less than 500 deliveries per year and had the highest utilisation of private GPs. It would be more costly for these hospitals to employ permanent doctors on the establishment and this model of public private contracting makes financial sense and provides access for women since the nearest regional hospital is roughly 150km from the district.

Considering broader implications of these findings for universal health care it is clear that experienced private GPs can play an important role in contributing much needed surgical and anaesthetic skills for safe caesarean deliveries in rural district hospitals. This is a model that could be considered for other provinces in SA and other low-and-middle income countries provided clinical decisions are driven by accepted public sector protocols and available support from specialists at referral regional hospitals can be ensured. Public-private partnerships have been criticized for being a more urban context option, but this is often on the assumption that the private providers will be obstetric specialists and this is the model that has been applied in settings such as India.[9 10 21] By contrast, in African countries such as Mozambique and Tanzania, clinical officers (healthcare providers trained to perform tasks usually undertaken by doctors) have been trained to perform caesarean deliveries. A meta-analysis reported no significant differences in outcomes of caesarean deliveries performed by clinical officers compared to medical doctors.[22] Different approaches to enable access to safe caesarean delivery are therefore needed for different contexts and experienced private GPs are a resource for rural district hospitals to consider. The sustainability of this model depends on the surgical and anaesthetic skills of future generations of GPs. The GPs skilled in obstetric surgery and anaesthesia in rural districts are mostly of an older age many of whom were previously district surgeons (a historical model of contracting private GPs within rural districts to provide curative primary care services within public sector health facilities).[23] More recently qualified GPs may have similar skills to their public sector



For peer review only - http://bmjopen.bmj.com/site/about/guidelines.xhtml

medical officer equivalents. The scale-up of this model therefore has implications for under-graduate basic surgical and anaesthetics skills training programmes.

**Limitations**

We collected obstetric data from registers and did not perform individual patient folder reviews. The proportion of caesarean delivery complications could have been higher if individual folders were reviewed. Post caesarean sepsis, thromboembolism, or postoperative bleeding that was detected in the postnatal ward, for example, could not be identified with the study methodology. Where no indication was listed in the theatre register for a caesarean delivery, this information would have been found had individual folders been reviewed. Furthermore, although a low proportion of women were referred following caesarean delivery, this group could have experienced morbidity or mortality but were not followed up to determine the reasons for referral or their outcomes at the regional referral hospital. We also did not collect data on neonatal outcomes which is an important indicator of the quality and safety of caesarean delivery.

**Conclusion**

The findings of this study suggest that private GPs can play an important role in filling gaps and expanding quality care in rural public facilities that have insufficient obstetric skills and expertise. In addition, provided that the broader systems and institutional framework to ensure compliance with clinical guidelines and protocols are in place, this can be done in a safe and effective manner, and could be seen as a prototype for NHI in rural contexts.

**Figure legends**

Figure 1: Private GP utilisation and caesarean delivery rate

Figure 2: Utilisation of private GPs and caesarean section profile and outcomes

**Acknowledgements** We thank all the health professionals who took time to participate in our interviews and to the hospital managers for facilitating access to the obstetric registers. We also thank Vishal Brijlal for his comments on a draft of this manuscript.



For peer review only - http://bmjopen.bmj.com/site/about/guidelines.xhtml

**Contributors** TD, GS, ED and SF conceptualised the study; TD, GS, ED and YB analysed the data; TD, GS, ED, AH, SF and YB contributed to the interpretation of the data, writing the paper and reviewing the content.

**Funding** The study was funded by the Bill and Melinda Gates Foundation (Grant Agreement ID INV-023276)

**Competing interests** None declared.
**Patient consent for publication** Not applicable.

**Ethics approval** Ethics approval was granted by the South African Medical Research Council Human Research Ethics Committee (approval number EC003-2/2021) and by the Western Cape Provincial Department of Health and Wellness (approval number WC_202103_017).

**Data availability** The aggregated routine data are available on request to the corresponding author.

For peer review only

## References


1. World Health Organisation. Trends in maternal mortality 2000-2017. Geneva: World Health Organisation, 2019.
2. National Department of Health. Saving Mothers 2017-2019. Seventh report on confidential enquiries into maternal deaths in South Africa. Pretoria: National Department of Health, 2020.
3. Solanki G, Fawcus S, Daviaud E. A cross sectional analytic study of modes of delivery and caesarean section rates in a private health insured South African population. *PloS one* 2019;14(6):e0219020. doi: 10.1371/journal.pone.0219020
4. Hoxha I, Fink G. Caesarean sections and health financing: a global analysis. *BMJ Open* 2021;11(5):e044383. doi: 10.1136/bmjopen-2020-044383 [published Online First: 2021/05/26]
5. Sobhy S, Arroyo-Manzano D, Murugesu N, et al. Maternal and perinatal mortality and complications associated with caesarean section in low-income and middle-income countries: a systematic review and meta-analysis. *The Lancet* 2019;393(10184):1973-82. doi: 10.1016/S0140-6736(18)32386-9
6. National Department of Health. Saving Mothers 2014-2016: Seventh triennial report on confidential enquiries into maternal deaths in South Africa. Pretoria: National Department of Health, 2017.
7. National Department of Health. National Health Insurance Bill. In: Health NDo, ed. Pretoria, 2019.
8. Amu H, Dickson KS, Kumi-Kyereme A, et al. Understanding variations in health insurance coverage in Ghana, Kenya, Nigeria, and Tanzania: Evidence from demographic and health surveys. *PloS one* 2018;13(8):e0201833. doi: 10.1371/journal.pone.0201833
9. Iyer V, Sidney K, Mehta R, et al. Availability and provision of emergency obstetric care under a public–private partnership in three districts of Gujarat, India: lessons for Universal Health Coverage. *BMJ Global Health* 2016;1(1):e000019. doi: 10.1136/bmjgh-2015-000019
10. Chaturvedi S, Randive B. Public private partnerships for emergency obstetric care: Lessons from Maharashtra. *Indian Journal of Community Medicine* 2011;36(1):21-26. doi: 10.4103/0970-0218.80788
11. Creswell John W, Plano Clark Vicki L. Choosing a Mixed Methods Design. Designing and Conducting Mixed Methods Research. 2 ed. Los Angeles, London, New Dehli: Sage 2011:53-106.
12. Green J, Thorogood, N. Qualitative Methods for Health Research. London: Sage Publications 2004.
13. Department of Health. A District Hospital Service Package for South Africa. Pretoria: Department of Health, 2002.
14. Gale NK, Heath G, Cameron E, et al. Using the framework method for the analysis of qualitative data in multi-disciplinary health research. *BMC Med Res Methodol* 2013;13:117. doi: 10.1186/1471-2288-13-117
15. Solanki GC, Cornell JE, Daviaud E, et al. Caesarean section rates in South Africa: A case study of the health systems challenges for the proposed National Health Insurance. *SAMJ* 2020;110(8)
16. World Health Organisation. WHO statement on caesarean section rates. Geneva: WHO, 2015.
17. Salazar M, Vora K, Sidney Annerstedt K, et al. Caesarean sections in the in the context of the Chiranjeevi Yojana public private partnership program to promote institutional birth in Gujarat, India; does the embedded disincentive for caesarean section work? *International journal for equity in health* 2019;18(1):17-17. doi: 10.1186/s12939-019-0922-5
18. Borrescio-Higa F, Valdés N. Publicly insured caesarean sections in private hospitals: a repeated cross-sectional analysis in Chile. *BMJ open* 2019;9(4):e024241-e41. doi: 10.1136/bmjopen-2018-024241
19. Fawcus S, Pattinson RC, Moodley J, et al. Maternal deaths from bleeding associated with caesarean delivery: A national emergency. *SAMJ* 2016;106(5)
20. Pattinson RC. Safety versus accessibility in maternal and perinatal care. *SAMJ* 2015;105(4)




For peer review only - http://bmjopen.bmj.com/site/about/guidelines.xhtml

21. Randive B, Chaturvedi S, Mistry N. Contracting in specialists for emergency obstetric care- does it work in rural India? *BMC Health Services Research* 2012;12(1):485. doi: 10.1186/1472-6963-12-485
22. Wilson A, Lissauer D, Thangaratinam S, et al. A comparison of clinical officers with medical doctors on outcomes of caesarean section in the developing world: meta-analysis of controlled studies. *Bmj* 2011;342:d2600. doi: 10.1136/bmj.d2600 [published Online First: 2011/05/17]
23. Kula N, Fryatt RJ. Public–private interactions on health in South Africa: opportunities for scaling up. *Health Policy and Planning* 2013;29(5):560-69. doi: 10.1093/heapol/czt042

For peer review only



For peer review only - http://bmjopen.bmj.com/site/about/guidelines.xhtml

Figure 1: Private GP utilisation and caesarean delivery rate

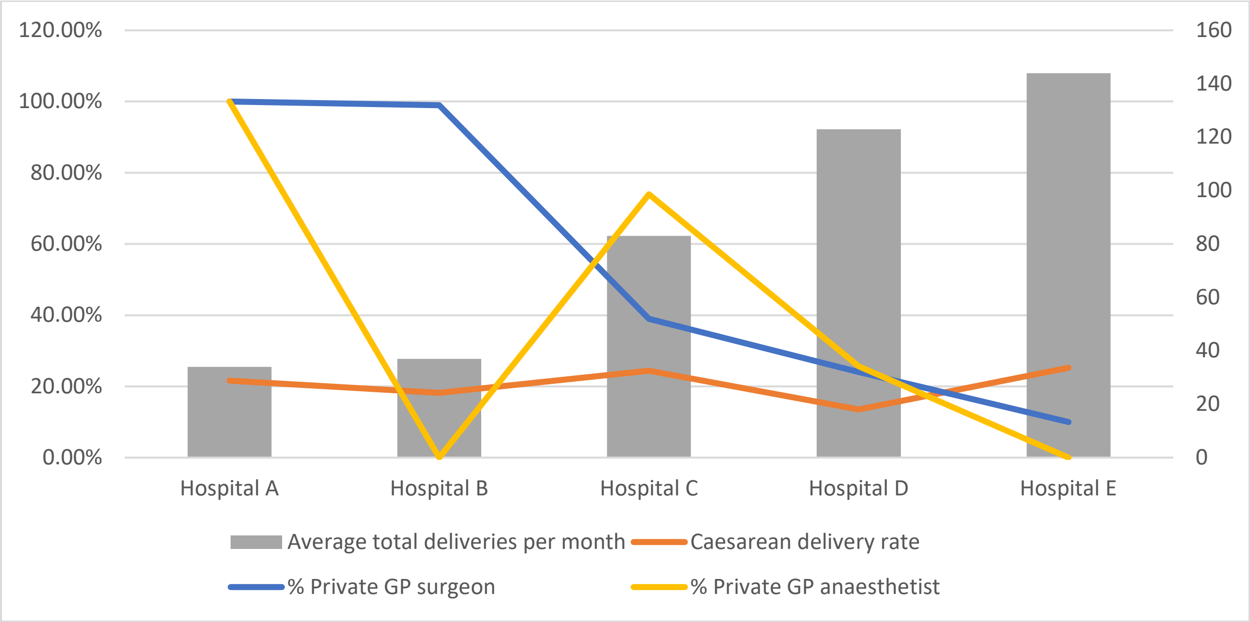

Figure 2: Utilisation of private GPs and caesarean delivery profile and outcomes

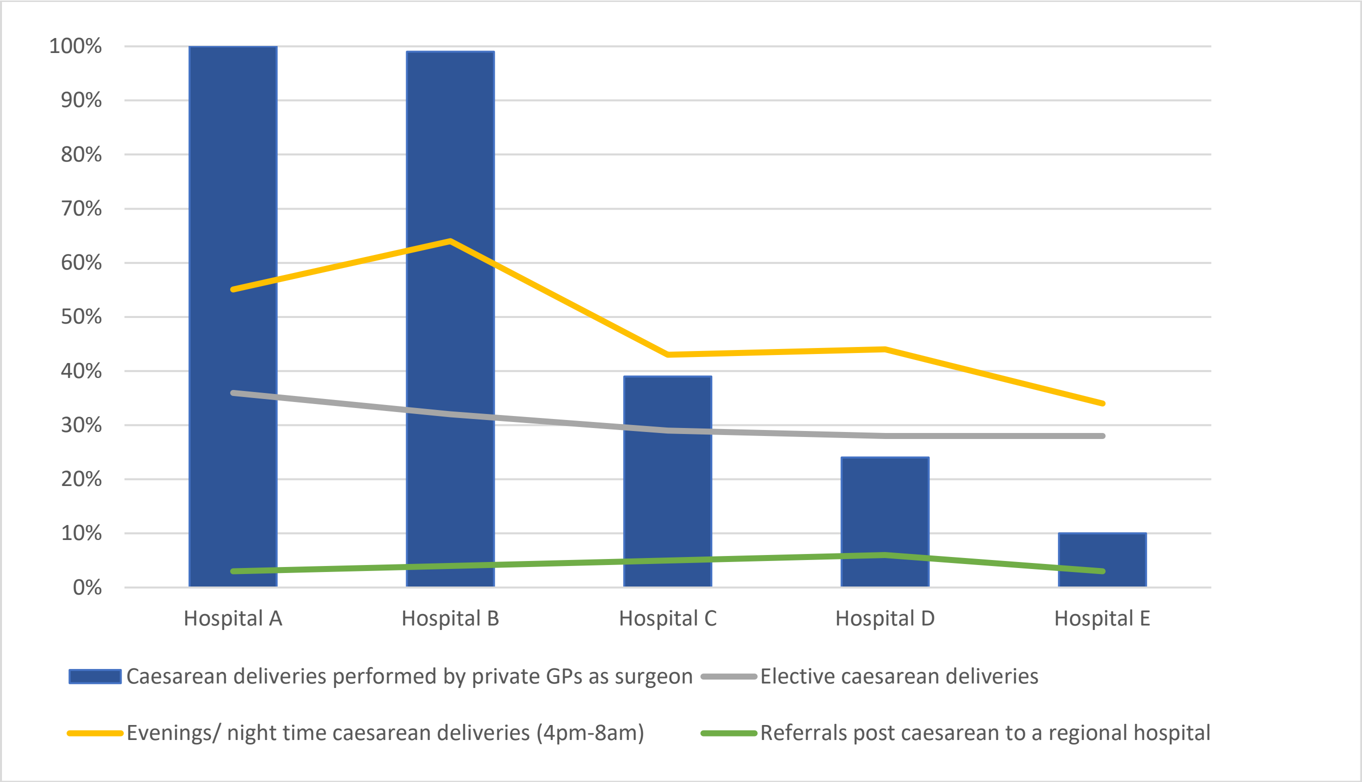


eview only

Supplementary tables

Supplementary table 1: Indications for emergency caesarean deliveries

| Indications * | Hospital A | | Hospital B | | Hospital C | | Hospital D | | Hospital E | | All Hospitals | |
|---|---|---|---|---|---|---|---|---|---|---|---|---|
| | N | % | N | % | N | % | N | % | N | % | N | % |
| Fetal distress/abnormal CTG | 15 | 26 | 28 | 51 | 77 | 45 | 79 | 55 | 155 | 49 | 354 | 48 |
| Prolonged labour/CPD | 6 | 10 | 10 | 18 | 24 | 14 | 18 | 12 | 37 | 12 | 95 | 13 |
| Previous CD (in labour) | 4 | 7 | 3 | 5 | 42 | 25 | 9 | 6 | 12 | 4 | 70 | 9 |
| Malpresentation (in labour) | 12 | 21 | 6 | 11 | 11 | 6 | 11 | 8 | 30 | 10 | 70 | 9 |
| Failed induction | 2 | 4 | 1 | 2 | 2 | 1 | 8 | 6 | 27 | 8 | 40 | 5 |
| placental abruption | 2 | 4 | 1 | 2 | 5 | 3 | 6 | 4 | 3 | 1 | 17 | 2 |
| Pre-eclampsia | 0 | 0 | 1 | 2 | 0 | 0 | 4 | 3 | 9 | 3 | 14 | 2 |
| PROM | 3 | 5 | 0 | 0 | 0 | 0 | 0 | 0 | 3 | 1 | 6 | 1 |
| Multiple pregnancy | 1 | 2 | 2 | 4 | 0 | 0 | 2 | 1 | 0 | 0 | 5 | 1 |
| APH | 0 | 0 | 0 | 0 | 0 | 0 | 1 | 1 | 2 | 1 | 3 | 0.4 |
| Failed VBAC | 0 | 0 | 0 | 0 | 0 | 0 | 0 | 0 | 0 | 0 | 0 | 0 |
| Big baby | 0 | 0 | 0 | 0 | 0 | 0 | 0 | 0 | 0 | 0 | 0 | 0 |
| Other | 2 | 4 | 3 | 5 | 4 | 2 | 4 | 3 | 17 | 5 | 30 | 4 |
| Number without listed indication | 10 | 17 | 0 | 0 | 6 | 4 | 1 | 1 | 20 | 6 | 37 | 5 |
| **Grand Total** | **57** | **100.00%** | **55** | **100.00%** | **171** | **100.00%** | **143** | **100.00%** | **315** | **100.00%** | **741** | **100.00%** |

CTG = cardiotocography ; CPD= cephalopelvic disproportion; APH = antepartum haemorrhage; CD = caesarean delivery; VBAC = vaginal birth after Caesarean;

PROM = premature rupture of membranes; APH = antepartum haemorrhage

*The indications for caesarean deliveries were those that were written in the theatre register so it was not possible to interrogate some of the indications such as PROM (prolonged rupture of membranes). According to Western Cape guidelines, caesarean delivery can be performed at District hospitals for suspected Big Baby. However, VBACs are not routinely performed at District hospitals and patients with pre-eclampsia would all be referred to regional or tertiary hospitals

For peer review only

Supplementary table 2: indications for elective caesarean sections

| Indications* | Hospital A | | Hospital B | | Hospital C | | Hospital D | | Hospital E | | All Hospitals | |
|---|---|---|---|---|---|---|---|---|---|---|---|---|
| | N | % | N | % | N | % | N | % | N | % | N | % |
| Previous CD | 5 | 16 | 16 | 61 | 50 | 70 | 40 | 71 | 66 | 55 | 177 | 58 |
| Malpresentation (breech/transverse) | 2 | 6 | 0 | 0 | 4 | 6 | 5 | 9 | 13 | 11 | 24 | 8 |
| HIV & unsuppressed VL | 1 | 3 | 2 | 8 | 0 | 0 | 0 | 0 | 8 | 7 | 11 | 3 |
| Multiple pregnancy | 0 | 0 | 0 | 0 | 1 | 1.4 | 1 | 2 | 3 | 2 | 5 | 2 |
| Big baby | 0 | 0 | 0 | 0 | 1 | 1.4 | 0 | 0 | 3 | 2 | 4 | 1 |
| Patient request | 0 | 0 | 0 | 0 | 0 | 0 | 0 | 0 | 2 | 2 | 2 | 1 |
| Diabetes & macrosomia | 0 | 0 | 0 | 0 | 0 | 0 | 0 | 0 | 1 | 1 | 1 | 0.3 |
| Obstructive vulvar warts | 0 | 0 | 0 | 0 | 0 | 0 | 0 | 0 | 0 | 0 | 0 | 0 |
| Other (genital herpes etc) | 1 | 3 | 1 | 4 | 7 | 10 | 1 | 2 | 10 | 8 | 20 | 7 |
| Number without listed indication | 23 | 72 | 7 | 27 | 8 | 11 | 9 | 16 | 15 | 12 | 62 | 20 |
| **Grand Total** | **32** | **100.00%** | **26** | **100.00%** | **71** | **100.00%** | **56** | **100.00%** | **121** | **100.00%** | **306** | **100.00%** |

CD = caesarean delivery; VL = viral load

*The indications for caesarean deliveries were those that were written in the theatre register so it was not possible to interrogate some of the indications. According to Western Cape guidelines, caesarean delivery can be performed at District hospitals for suspected Big Baby.

For peer review only

STROBE Statement—checklist of items that should be included in reports of observational studies

| | Item No. | Recommendation | Page No. | Relevant text from manuscript |
|---|---|---|---|---|
| **Title and abstract** | 1 | (*a*) Indicate the study's design with a commonly used term in the title or the abstract | 1 | |
| | | (*b*) Provide in the abstract an informative and balanced summary of what was done and what was found | 2 | |
| **Introduction** | | | | |
| Background/rationale | 2 | Explain the scientific background and rationale for the investigation being reported | 4&5 | |
| Objectives | 3 | State specific objectives, including any prespecified hypotheses | 5 | |
| **Methods** | | | | |
| Study design | 4 | Present key elements of study design early in the paper | 6&7 | |
| Setting | 5 | Describe the setting, locations, and relevant dates, including periods of recruitment, exposure, follow-up, and data collection | 6&7 | |
| Participants | 6 | (*a*) *Cohort study*—Give the eligibility criteria, and the sources and methods of selection of participants. Describe methods of follow-up<br>*Case-control study*—Give the eligibility criteria, and the sources and methods of case ascertainment and control selection. Give the rationale for the choice of cases and controls<br>*Cross-sectional study*—Give the eligibility criteria, and the sources and methods of selection of participants | 7 | |
| | | (*b*) *Cohort study*—For matched studies, give matching criteria and number of exposed and unexposed<br>*Case-control study*—For matched studies, give matching criteria and the number of controls per case | | |
| Variables | 7 | Clearly define all outcomes, exposures, predictors, potential confounders, and effect modifiers. Give diagnostic criteria, if applicable | 7 | |
| Data sources/ measurement | 8* | For each variable of interest, give sources of data and details of methods of assessment (measurement). Describe comparability of assessment methods if there is more than one group | 7 | |
| Bias | 9 | Describe any efforts to address potential sources of bias | 21 | |
| Study size | 10 | Explain how the study size was arrived at | 7 | |

Continued on next page



For peer review only - http://bmjopen.bmj.com/site/about/guidelines.xhtml

| | | | |
|---|---|---|---|
| Quantitative variables | 11 | Explain how quantitative variables were handled in the analyses. If applicable, describe which groupings were chosen and why | 9 |
| Statistical methods | 12 | (*a*) Describe all statistical methods, including those used to control for confounding | 9 |
| | | (*b*) Describe any methods used to examine subgroups and interactions | 9 |
| | | (*c*) Explain how missing data were addressed | |
| | | (*d*) *Cohort study*—If applicable, explain how loss to follow-up was addressed<br>*Case-control study*—If applicable, explain how matching of cases and controls was addressed<br>*Cross-sectional study*—If applicable, describe analytical methods taking account of sampling strategy | |
| | | (*e*) Describe any sensitivity analyses | |
| **Results** | | | |
| Participants | 13* | (a) Report numbers of individuals at each stage of study—eg numbers potentially eligible, examined for eligibility, confirmed eligible, included in the study, completing follow-up, and analysed | table 2 |
| | | (b) Give reasons for non-participation at each stage | |
| | | (c) Consider use of a flow diagram | |
| Descriptive data | 14* | (a) Give characteristics of study participants (eg demographic, clinical, social) and information on exposures and potential confounders | Table 1 |
| | | (b) Indicate number of participants with missing data for each variable of interest | |
| | | (c) *Cohort study*—Summarise follow-up time (eg, average and total amount) | |
| Outcome data | 15* | *Cohort study*—Report numbers of outcome events or summary measures over time | |
| | | *Case-control study*—Report numbers in each exposure category, or summary measures of exposure | |
| | | *Cross-sectional study*—Report numbers of outcome events or summary measures | tables 2 and 3 |
| Main results | 16 | (*a*) Give unadjusted estimates and, if applicable, confounder-adjusted estimates and their precision (eg, 95% confidence interval). Make clear which confounders were adjusted for and why they were included | pages 10-17 |
| | | (*b*) Report category boundaries when continuous variables were categorized | table 1 |
| | | (*c*) If relevant, consider translating estimates of relative risk into absolute risk for a meaningful time period | |

Continued on next page

For peer review only



For peer review only - http://bmjopen.bmj.com/site/about/guidelines.xhtml

| | | | |
|---|---|---|---|
| Other analyses | 17 | Report other analyses done—eg analyses of subgroups and interactions, and sensitivity analyses | |
| **Discussion** | | | |
| Key results | 18 | Summarise key results with reference to study objectives | 18-20 |
| Limitations | 19 | Discuss limitations of the study, taking into account sources of potential bias or imprecision. Discuss both direction and magnitude of any potential bias | 21 |
| Interpretation | 20 | Give a cautious overall interpretation of results considering objectives, limitations, multiplicity of analyses, results from similar studies, and other relevant evidence | 21 |
| Generalisability | 21 | Discuss the generalisability (external validity) of the study results | 21 |
| **Other information** | | | |
| Funding | 22 | Give the source of funding and the role of the funders for the present study and, if applicable, for the original study on which the present article is based | 22 |

*Give information separately for cases and controls in case-control studies and, if applicable, for exposed and unexposed groups in cohort and cross-sectional studies.

**Note:** An Explanation and Elaboration article discusses each checklist item and gives methodological background and published examples of transparent reporting. The STROBE checklist is best used in conjunction with this article (freely available on the Web sites of PLoS Medicine at http://www.plosmedicine.org/, Annals of Internal Medicine at http://www.annals.org/, and Epidemiology at http://www.epidem.com/). Information on the STROBE Initiative is available at www.strobe-statement.org.

For peer review only

# Standards for Reporting Qualitative Research (SRQR)*

http://www.equator-network.org/reporting-guidelines/srqr/

## Title and abstract

| | Page/line no(s). |
|---|---|
| **Title** - Concise description of the nature and topic of the study Identifying the study as qualitative or indicating the approach (e.g., ethnography, grounded theory) or data collection methods (e.g., interview, focus group) is recommended | 1 |
| **Abstract** - Summary of key elements of the study using the abstract format of the intended publication; typically includes background, purpose, methods, results, and conclusions | 2 |

## Introduction

| | |
|---|---|
| **Problem formulation** - Description and significance of the problem/phenomenon studied; review of relevant theory and empirical work; problem statement | 5 |
| **Purpose or research question** - Purpose of the study and specific objectives or questions | 5 |

## Methods

| | |
|---|---|
| **Qualitative approach and research paradigm** - Qualitative approach (e.g., ethnography, grounded theory, case study, phenomenology, narrative research) and guiding theory if appropriate; identifying the research paradigm (e.g., postpositivist, constructivist/ interpretivist) is also recommended; rationale** | 6 |
| **Researcher characteristics and reflexivity** - Researchers' characteristics that may influence the research, including personal attributes, qualifications/experience, relationship with participants, assumptions, and/or presuppositions; potential or actual interaction between researchers' characteristics and the research questions, approach, methods, results, and/or transferability | 8 |
| **Context** - Setting/site and salient contextual factors; rationale** | 6/7 |
| **Sampling strategy** - How and why research participants, documents, or events were selected; criteria for deciding when no further sampling was necessary (e.g., sampling saturation); rationale** | 8 |
| **Ethical issues pertaining to human subjects** - Documentation of approval by an appropriate ethics review board and participant consent, or explanation for lack thereof; other confidentiality and data security issues | 9/10 |
| **Data collection methods** - Types of data collected; details of data collection procedures including (as appropriate) start and stop dates of data collection and analysis, iterative process, triangulation of sources/methods, and modification of procedures in response to evolving study findings; rationale** | 8 |



For peer review only

For peer review only - http://bmjopen.bmj.com/site/about/guidelines.xhtml

| | |
|---|---|
| **Data collection instruments and technologies** - Description of instruments (e.g., interview guides, questionnaires) and devices (e.g., audio recorders) used for data collection; if/how the instrument(s) changed over the course of the study | 8 |
| **Units of study** - Number and relevant characteristics of participants, documents, or events included in the study; level of participation (could be reported in results) | 8 |
| **Data processing** - Methods for processing data prior to and during analysis, including transcription, data entry, data management and security, verification of data integrity, data coding, and anonymization/de-identification of excerpts | 9 |
| **Data analysis** - Process by which inferences, themes, etc., were identified and developed, including the researchers involved in data analysis; usually references a specific paradigm or approach; rationale** | 9 |
| **Techniques to enhance trustworthiness** - Techniques to enhance trustworthiness and credibility of data analysis (e.g., member checking, audit trail, triangulation); rationale** | 9 |

**Results/findings**

| | |
|---|---|
| **Synthesis and interpretation** - Main findings (e.g., interpretations, inferences, and themes); might include development of a theory or model, or integration with prior research or theory | 10-17 |
| **Links to empirical data** - Evidence (e.g., quotes, field notes, text excerpts, photographs) to substantiate analytic findings | 10-17 |

**Discussion**

| | |
|---|---|
| **Integration with prior work, implications, transferability, and contribution(s) to the field -** Short summary of main findings; explanation of how findings and conclusions connect to, support, elaborate on, or challenge conclusions of earlier scholarship; discussion of scope of application/generalizability; identification of unique contribution(s) to scholarship in a discipline or field | 18-20 |
| **Limitations** - Trustworthiness and limitations of findings | 21 |

**Other**

| | |
|---|---|
| **Conflicts of interest** - Potential sources of influence or perceived influence on study conduct and conclusions; how these were managed | 22 |
| **Funding** - Sources of funding and other support; role of funders in data collection, interpretation, and reporting | 22 |

*The authors created the SRQR by searching the literature to identify guidelines, reporting standards, and critical appraisal criteria for qualitative research; reviewing the reference lists of retrieved sources; and contacting experts to gain feedback. The SRQR aims to improve the transparency of all aspects of qualitative research by providing clear standards for reporting qualitative research.

For peer review only

**The rationale should briefly discuss the justification for choosing that theory, approach, method, or technique rather than other options available, the assumptions and limitations implicit in those choices, and how those choices influence study conclusions and transferability. As appropriate, the rationale for several items might be discussed together.

**Reference:**

O'Brien BC, Harris IB, Beckman TJ, Reed DA, Cook DA. **Standards for reporting qualitative research: a synthesis of recommendations.** *Academic Medicine*, Vol. 89, No. 9 / Sept 2014 DOI: 10.1097/ACM.0000000000000388

For peer review only

BMJ Open

# Utilisation of private general practitioners to provide caesarean deliveries in five rural district public hospitals in South Africa: a mixed-method study

| | |
|---|---|
| Journal: | *BMJ Open* |
| Manuscript ID | bmjopen-2022-067663.R2 |
| Article Type: | Original research |
| Date Submitted by the Author: | 13-Feb-2023 |
| Complete List of Authors: | Doherty, Tanya; South African Medical Research Council, Health Systems Research Unit<br>Solanki, Geetesh; South African Medical Research Council, HSRU<br>Daviaud, Emmanuelle; South African Medical Research Council<br>Bartmann, Yvandi; South African Medical Research Council<br>Hawkridge, Anthony; Western Cape Department of Health<br>Fawcus, Sue ; University of Cape Town Faculty of Health Sciences |
| <b>Primary Subject Heading</b>: | Obstetrics and gynaecology |
| Secondary Subject Heading: | Health services research |
| Keywords: | OBSTETRICS, PUBLIC HEALTH, Organisation of health services < HEALTH SERVICES ADMINISTRATION & MANAGEMENT |
| | |

SCHOLARONE™
Manuscripts

*I, the Submitting Author has the right to grant and does grant on behalf of all authors of the Work (as defined in the below author licence), an exclusive licence and/or a non-exclusive licence for contributions from authors who are: i) UK Crown employees; ii) where BMJ has agreed a CC-BY licence shall apply, and/or iii) in accordance with the terms applicable for US Federal Government officers or employees acting as part of their official duties; on a worldwide, perpetual, irrevocable, royalty-free basis to BMJ Publishing Group Ltd ("BMJ") its licensees and where the relevant Journal is co-owned by BMJ to the co-owners of the Journal, to publish the Work in this journal and any other BMJ products and to exploit all rights, as set out in our licence.*

*The Submitting Author accepts and understands that any supply made under these terms is made by BMJ to the Submitting Author unless you are acting as an employee on behalf of your employer or a postgraduate student of an affiliated institution which is paying any applicable article publishing charge ("APC") for Open Access articles. Where the Submitting Author wishes to make the Work available on an Open Access basis (and intends to pay the relevant APC), the terms of reuse of such Open Access shall be governed by a Creative Commons licence – details of these licences and which Creative Commons licence will apply to this Work are set out in our licence referred to above.*

*Other than as permitted in any relevant BMJ Author's Self Archiving Policies, I confirm this Work has not been accepted for publication elsewhere, is not being considered for publication elsewhere and does not duplicate material already published. I confirm all authors consent to publication of this Work and authorise the granting of this licence.*

For peer review only

# Utilisation of private general practitioners to provide caesarean deliveries in five rural district public hospitals in South Africa: a mixed-method study

Authors

Tanya Doherty[1,2], Geetesh Solanki[1], Emmanuelle Daviaud[1], Yvandi Bartmann[1], Anthony Hawkridge[3], Sue Fawcus[4]

Affiliations

[1] Health Systems Research Unit, South African Medical Research Council, Cape Town, South Africa

[2] Department of Paediatrics and Child Health, University of Cape Town, Cape Town, South Africa

[3] Swartland Subdistrict, Western Cape Department of Health, Malmesbury, South Africa

[4] Department of Obstetrics and Gynaecology, University of Cape Town, Cape Town, South Africa

**Correspondence to** Prof Tanya Doherty tanya.doherty@mrc.ac.za

For peer review only

Abstract

Objective

Researching how public-private engagements may promote universal access to safe obstetric care including caesarean delivery is essential. The aim of this research was to document the utilisation of private general practitioners (GPs) contracted to provide caesarean delivery services in five rural district hospitals in the Western Cape, the profile and outcomes of caesarean deliveries. We also describe stakeholder experiences of these arrangements in order to inform potential models of public private contracting for obstetric services.

Methods

We utilised a mixed-method study design to describe rural district hospitals' utilization of private GP contracting for caesarean deliveries. Between April 2021 and March 2022 we collated routine data from delivery and theatre registers to capture the profile of deliveries and maternal outcomes. We conducted 23 semi-structured qualitative interviews with district managers, hospital employed doctors and private GPs to explore their experiences of the contracting arrangements.

Results

The use of private GPs as surgeon or anaesthetist for caesarean deliveries differed widely across the hospitals. Overall, the utilisation of private GPs for anaesthetics was similar (29% of all caesarean deliveries) to the utilisation of private GPs as surgeons (33% of all caesarean deliveries). The proportion of caesarean deliveries undertaken by private GPs as the primary surgeon was inversely related to size of hospital and mean monthly deliveries. Adverse outcomes following a caesarean delivery were rare. Qualitative data provided insights into contributions made by private GPs and the contracting models which did not incentivize over-servicing.

Conclusion

The findings of this study suggest that private GPs can play an important role in filling gaps and expanding quality care in rural public facilities that have insufficient obstetric skills and expertise. Different approaches to enable access to safe caesarean delivery are needed for different contexts and contracting with experienced private is one resource for rural district hospitals to consider.



For peer review only - http://bmjopen.bmj.com/site/about/guidelines.xhtml

**Strengths and Limitations**

- This is one of the first studies to describe utilisation of private GPs to support caesarean deliveries within rural district hospital settings in a low-and-middle income country context.
- A mixed-method study design including routine obstetric data and qualitative interviews provides a robust description of experiences with this public-private model.
- We collated obstetric data from registers and did not perform individual patient folder reviews. The proportion of caesarean delivery complications could have been higher if individual folders were reviewed.
- We did not collect data on neonatal outcomes which is also an important indicator of the quality and safety of caesarean delivery.

For peer review only

## Introduction

Maternal and child health is a major public health concern in South Africa (SA). Whilst there has been progress in reducing maternal mortality to 119 per 100 000 live births,[1] there remain huge disparities between public and private sectors in the distribution of obstetric care providers, models of care and outcomes.[2] The caesarean delivery rate in the public sector is 28%[2] compared to 74% in the private sector.[3] A global analysis exploring the relationship between health financing indicators and caesarean delivery rates found that in 2018 there were a total of 8.8 million unnecessary caesarean deliveries globally, two-thirds of which occurred in upper middle-income countries.[4] The study reported that a 10 per cent increase in voluntary health insurance was associated with a 4 per cent increase in excess caesarean deliveries, defined as caesarean delivery proportions above the global target of 19%.[4]

In line with patterns seen in other low-and-middle-income countries,[5] the case fatality rate (CFR) for caesarean deliveries in the SA public sector is three times higher than for vaginal delivery and 27% of the caesarean delivery related CFR is associated with haemorrhage.[2] Caesarean delivery CFR and hemorrhage rates are not routinely measured in the private sector so such data is not available. The 2014-2016 Saving Mothers report for SA described a review of maternal deaths in the private sector. The maternal mortality rate was 45 per 100,000 live births (one third that of the national SA rate at the time) with a similar proportion of deaths from obstetric hemorrhage (19%) of which 17% were from caesarean delivery associated bleeding.[6] Improved clinical governance and skills development in the public sector are critical to reducing this disparity, but these efforts could be complemented by the engagement of health professionals with obstetric skills in the private sector.

In efforts towards achieving Universal Health Coverage (UHC), SA is in the process of developing policy to allow implementation of a National Health Insurance (NHI). The NHI Bill[7] proposes that NHI will be a strategic purchaser of health care services for the entire SA population. The proposed NHI will contract for obstetric services from both public and private providers. A key challenge in SA regardless of when and in what form NHI is implemented, is to develop contracting models that allow public and private resources to be mobilized to the benefit of the entire population. This is particularly important for rural district hospitals where surgical skills and supervision may be insufficient to meet the service requirements for obstetric care, and specialist services are not readily available in the public or private sectors.



For peer review only - http://bmjopen.bmj.com/site/about/guidelines.xhtml

However, attempts to harness the resources of the private sector to service the needs of the broader SA population would need to address the challenge of ensuring that the care provided is appropriate and patterns of inappropriate care, including the high caesarean delivery rate of the private sector[3], is not reproduced for the entire population. The development of contracting models that would best mitigate these risks is critical in delivering UHC.

Researching and documenting how private sector resources can be harnessed to support the public health system is urgently needed now, in the transition, and full implementation phases of NHI in SA and also to inform developments in other African countries that are implementing similar national health insurance financing arrangements. These include countries such as Ghana, Kenya, Nigeria and Tanzania.[8] There are some lessons to be learnt from public-private contracting for obstetric services in India where states have implemented different models of contracting arrangements to increase access to obstetric care[9]. In some states private obstetricians are contracted in to public facilities to increase skills and human resources while in other states costs for services are subsidized within private facilities.[10]

Research to understand whether public-private engagements could reduce inequities and promote universal access to safe obstetric care including caesarean delivery is essential. Understanding the nature of public-private engagements in the area of obstetric care is particularly important because caesarean delivery is the most commonly performed surgical procedure and the findings may also be useful in informing strategies for other medical and surgical disciplines where similar patterns of under servicing and over-servicing in the public vs. private sector may occur.

We carried out research to document the utilisation of private general practitioners (GPs) contracted to provide caesarean delivery services in five rural district hospitals in the Western Cape and to qualitatively describe the experiences and perspectives of managers and doctors involved with a view to informing the development of public private arrangements for improving obstetric and maternal outcomes in rural areas in South Africa and LMICs more broadly. The aim of this paper is to document our findings on the utilisation of the GP's, the profile and outcomes of caesarean deliveries. We also describe stakeholder experiences of these arrangements in order to inform the development of models of public private contracting for obstetric services.



For peer review only - http://bmjopen.bmj.com/site/about/guidelines.xhtml

## Methods

Study design

We undertook descriptive health systems research utilising a mixed-method study design to describe rural district hospitals' utilization of private GP contracting for caesarean deliveries in the Western Cape province and the profile and maternal outcomes of these deliveries in five hospitals. We chose a mixed-method study design incorporating both quantitative and qualitative data collection as this approach is well suited to applied health systems research.[11 12]

Study setting

The setting for this research was the Western Cape province where existing public-private contracting for caesarean delivery services was occurring due to human resource shortages in rural district hospitals. Five rural district hospitals within one rural district were chosen following engagement with provincial managers and obstetric clinical managers.

In South Africa, women with low-risk pregnancies receive antenatal care at primary care clinics and community health centres. District hospitals provide level 1 (generalist) services to in-patients and outpatients including obstetric care for women with low-risk pregnancies. District hospitals have between 30 and 200 beds, a 24-hour emergency service and an operating theatre. Generalists (medical officers) provide the services together with nursing staff and allied health professionals, some district hospitals have specialist family physicians serving as clinical managers but there are no obstetric or anaesthetic specialists at district hospital level. Most district hospitals also have community service doctors. These are doctors who have completed a two-year internship and are required to complete a further one year of community service.[13] None of the five hospitals had newly qualified intern medical doctors who are generally not placed within district hospitals.

For obstetric services at district hospital level normal vaginal deliveries are performed by midwives, assisted vaginal deliveries are performed by advanced midwives or medical officers and caesarean deliveries (surgery and anaesthesia) are performed by medical officers. Pregnant women with pre-existing morbidities such as diabetes, autoimmune disorders, thyroid disease, and cardiac disease or obstetric complications such as anticipated preterm delivery, suspected intra-uterine growth restrictions, pre-eclampsia, placenta praevia, abruptio placentae, multiple pregnancy, two previous



For peer review only - http://bmjopen.bmj.com/site/about/guidelines.xhtml

caesarean sections, Body Mass Index over 35-40 kgs/m², and severe anaemia are referred for delivery to a secondary or tertiary level hospital.

Public health facilities are permitted to contract the services of private providers where needed. There are three mechanisms by which private providers can be contracted to the public service: through a locum agency, through a sessional contract which is limited to a maximum of 39 hours per month or as a service provider in response to a tender for specific services. In all three contracting models the remuneration is time-based and not related to the number of patients or theatre cases performed. In the case of obstetric services private providers are mainly utilized for theatre services either as a GP surgeon or GP anaesthetist to undertake caesarean deliveries or for obstetric surgery including ectopic pregnancy, termination of pregnancy and dilatation and curettage following spontaneous miscarriage. They may also be called for an assisted delivery if the establishment doctor is unable to manage a complicated delivery. For GPs contracted through a sessional contract, medico-legal indemnity is provided by the state but for those contracted as locums or through a service provider tender they are required to have their own medico-legal indemnity cover. In these five hospitals the private GPs did not have medical indemnity for private obstetric practice and only performed caesarean deliveries during their public sector contracted time.

Quantitative data collection

For a period of 12 months (1 April 2021 – 31 March 2022) we collated quantitative obstetric clinical data from all five participating hospitals. Clinical outcomes were captured from the delivery register, theatre register and obstetric transfer book. Outcomes that could be collated from these sources without individual patient folder review included: mode of delivery and caesarean delivery complications (maternal death, postpartum haemorrhage (PPH) and referral to a regional hospital). Other procedures performed at or after a caesarean delivery were also captured as a measure of complications (hysterectomy, B-lynch suture and re-look laparotomy). For each caesarean delivery we captured whether the surgeon and anaesthetist was a hospital employed medical officer or a private GP, the type of anaesthetic given, whether the procedure was classified as an emergency or elective and whether it was performed during daytime or evening/night time (4pm – 8am, Monday- Sunday).



For peer review only - http://bmjopen.bmj.com/site/about/guidelines.xhtml

Clinical data was collated from registers by research nurses recruited for the purpose of the study. The monthly data was entered into a pre-set excel spreadsheet.

Qualitative data collection

Semi-structured interview guides were developed, one for private providers and one for public providers. Qualitative interviews were undertaken by three of the investigators (TD, GS and ED) at the hospitals and district office. The investigators who conducted interviews are all senior researchers with experience in qualitative interviewing. Two were health economists and one a health systems researcher. They had no prior relationships with any of the interviewees. All interviews were conducted in a private consulting room or office and were undertaken in English.

Interviews were undertaken with private GPs who had entered into public service contracts with each of the five hospitals to explore their perceptions of undertaking work in the public sector and the role they played. At each hospital we also interviewed the hospital managers and public providers (doctors) to explore their perceptions of the public/ private interface including clinical decision making, private GP availability and remuneration models. Types of questions asked to private GPs included: '' What has been your experience of working in government hospitals? and 'How do you balance your time between private practice and government hospital work?''; types of questions asked to district managers and government doctors included: '' What services are private GPs contracted to provide?, ''How are decisions about caesarean delivery made within the clinical team? , ''How are private GPs remunerated for their time by the public sector?'' During interviews we also collected information on the total number of hours per month each GP was contracted to the public hospital and used this information to calculate the number of fulltime equivalent doctor posts the GPs contributed at each hospital. For example, in hospital C there were 5 private GPs each contracted for 25 hours per week. A public sector doctor works 56 hours per week therefore in this hospital the combined private GP hours are equivalent to 2.2 fulltime equivalent establishment doctors.

We undertook a total of 23 semi-structured qualitative interviews; including 3 district managers (2 male, 1 female), twelve private GPs (7 male, 5 female) and 8 government employed doctors (4 male, 4 female).



For peer review only - http://bmjopen.bmj.com/site/about/guidelines.xhtml

Quantitative data analysis

Quantitative data from hospital records was analysed in excel using simple descriptive mean and standard deviation (mean monthly number of deliveries) and proportions (caesarean delivery rate, deliveries undertaken by a private GP, elective caesarean deliveries and caesarean deliveries occurring during daytime or evening/ night time hours) stratifying by hospital over the 12 month data collation period.

We describe the proportion of total annual caesarean deliveries at each hospital undertaken by private GPs as surgeon or anaesthetist and explore the relationship between type of provider and the caesarean delivery profile (elective vs emergency and timing in terms of day time hours or during the evening/night) and adverse outcomes (referral to a regional hospital following a caesarean delivery, maternal death, postpartum haemorrhage and other procedures performed at caesarean deliveries).

Qualitative data analysis

Qualitative interviews were digitally recorded and transcribed. A framework analysis[14] approach was applied which is well suited to implementation research designs. We drew on a framework previously developed by members of our research team which outlines drivers, challenges and required action for obstetric care in preparing for NHI.[15] The interviews were read and re-read by members of the team to familiarise ourselves with the content. Emerging categories and major themes were identified and mapped against the framework and quantitative findings. Five members of the research team (TD, ED, GS, SF and YB) read transcripts and met to discuss emerging categories and major themes. The qualitative data enhance contextual understanding of the quantitative outcomes related to CS rates and outcomes by shedding light on provider experiences and perceptions of aspects such as workload and remuneration.

Ethical considerations

Ethics approval was granted by the South African Medical Research Council Human Research Ethics Committee (approval number EC003-2/2021) and permission was also granted by the Western Cape Provincial Department of Health and Wellness (approval number WC_202103_017). No personal identifying information was captured from hospital records, only aggregated summary information. All health professionals who were approached to participate in an interview were read an information sheet outlining the expectations in terms of length of the discussion, the voluntary nature of the



For peer review only - http://bmjopen.bmj.com/site/about/guidelines.xhtml

participation and measures to ensure confidentiality. Health professionals who agreed to participate signed an informed consent form.

**Patient and public involvement** Patients and/or the public were not involved in the design, or conduct, of this research. We will be disseminating the findings including through presentations at each of the participating hospitals which will be targeted at stakeholder groups including patients and members of the public.

## Results

From the analysis of the qualitative interviews four main themes were identified which included: (1) the role played by private GPs contracted to district hospitals; (2) the nature of clinical decision making regarding caesarean delivery; (3) balancing work between private practice and public sector and (4) perspectives on remuneration models. These results are reported in narrative form, as quotes, woven within the quantitative results below.

### Hospital characteristics and utilisation of private GPs for caesarean deliveries

The five district hospitals ranged in size from 35 beds in hospital A to 118 beds in hospital E. Over the period 1 April 2021 to 31 March 2022, the average number of monthly deliveries ranged from 34 in hospital A to 144 in hospital E. None of the hospitals has primary care clinics in its catchment areas that perform vaginal deliveries other than in the case of emergencies and only during daytime hours. All five hospitals have some involvement of private GPs to assist with the obstetric service but the level of utilisation of their services differed greatly across the hospitals. In hospital A there are no government employed doctors and the hospital is run entirely by a private GP practice consisting of 5 doctors who provide 24-hour cover to the hospital. Across the other hospitals the number of contracted GPs ranged from 1 in hospital E to 5 in hospital C but this translated into a maximum of 2 full time equivalent doctors in hospital C and 0.2 in hospital E (table 1).



For peer review only - http://bmjopen.bmj.com/site/about/guidelines.xhtml

Table 1: Characteristics of participating hospitals

| Hospital | Hospital A | Hospital B | Hospital C | Hospital D | Hospital E |
|---|---|---|---|---|---|
| Number of beds | 35 | 50 | 75 | 85 | 118 |
| Number of maternity beds (antenatal, labour and postnatal) | 8 | 10 | 12 | 14 | 25 |
| Number of clinics in the hospital catchment that perform vaginal deliveries | 0 | 5 clinics that can do emergency vaginal deliveries if required, no 24 hour service | 0 | 1 community day clinic, 4 clinics and 6 satellite services, none offer 24 hour service, can do emergency vaginal deliveries if required | 10 clinics that can do emergency vaginal deliveries if required, no 24 hour service |
| Mean monthly number of deliveries (SD) | 34 (7.2) | 37 (9.7) | 83 (5.2) | 123 (9.8) | 144 (18.3) |
| Number of fulltime hospital employed doctors | 0 | 6 | 6 | 10 (2 of which are specialist family physicians) | 13 |
| Number of community service doctors included in the total medical staff | 0 | 4 | 2 | 4 | 5 |
| Number of privately contracted GPs to assist with the obstetric service | One practice consisting of 5 GPs providing a 24- hour service undertaking both surgery and anaesthesia | 2 GPs undertaking surgery | 5 (2 for anaesthetics and 3 for surgery) | 4 (2 surgical, 2 anaesthetic); 1 GP has a post-graduate diploma in anaesthetics, 1 GP has a post-graduate diploma in obstetrics, 1 GP is a specialist family physician | 1 GP doing surgery only |
| Number of full-time equivalent private GPs | 0.9 | 0.3 | 2.2 | 0.9 | 0.2 |

SD= standard deviation



For peer review only - http://bmjopen.bmj.com/site/about/guidelines.xhtml

Supplementary table 1 shows the profile of deliveries occurring in the hospitals over the 12-month study period. The proportion of normal vaginal deliveries ranged from 74% in hospitals C and E to 85% in hospital D. Assisted vaginal deliveries were uncommon (<3%) across all five hospitals. The caesarean delivery rate ranged from 14% in hospital D to 25% in hospital E. Spinal anaesthesia was used in 95% of all cases.

The use of private GPs as surgeon or anaesthetist for caesarean deliveries differed widely across the five hospitals. Overall, the utilisation of private GPs for anaesthetics was similar to (29% of all caesarean deliveries) the utilisation of private GPs as surgeons (33% of all caesarean deliveries) although in hospital C more private GPs were utilised for anaesthetics than for surgery (Supplementary table 1). Interviews with private GPs and hospital employed doctors spoke to the role that private GPs play in district hospitals (theme 1) and revealed that the skills shortage was perceived to be surgical rather than anaesthetic, and that private GPs make a contribution to training community service doctors in surgical skills for caesarean deliveries as these doctors explained:

*I'm doing more the cutting side. So, I mean, the comserves (community service doctor) can't cut. I am always cutting. There's one comserve on and it's me. So, we cut with the sister (Private GP, Hospital B).*

*So, in a month I must train all the community service doctors to be safe to do caesarean deliveries and ectopics which is not an easy undertaking (Clinical manager, Hospital D).*

*That is just the GP that is usually the primary surgeon. I am more of the assistant. I'm still learning to do caesars on my own. So, it's usually just the GPs who are doing the primary surgery. On the anaesthetic side there's another GP that is on call. We're basically an assistant. I'm still learning so I mostly doing primi-gravida's at the moment (Year 1 medical officer, Hospital C).*

*I'm on call every second day of my life and every second weekend of my life because we, as the two of us (private GPs), are the only people that can do caesarean deliveries. The rest of the people are either young MOs with little experience or, community service doctors that cannot do that (Private GP, Hospital B).*



For peer review only - http://bmjopen.bmj.com/site/about/guidelines.xhtml

The proportion of caesarean deliveries undertaken by private GPs as the primary surgeon was inversely related to size of hospital and total average monthly deliveries (figure 1). The two smallest hospitals were most reliant on private GPs to perform caesarean deliveries and as size of hospital increased fewer caesarean deliveries were undertaken by private GPs. The caesarean delivery rate was not higher in hospitals where private GPs carried out a higher proportion of caesarean procedures (figure 1). During interviews the reasons given for this were that it is generally the hospital-based staff who make the decision about whether a caesarean delivery is required, and the private GP is not involved in that decision. In hospital A, which is run entirely by private GPs the midwives managing the labour ward assess progress of labour and call the GPs from their practice if they assess that a caesarean is required. The following quotes illustrate the decision-making around caesarean deliveries (theme 2):

*The contract that I have is explicitly for theatre work only. Nothing else. I don't go into labour ward, maternity ward. I get called and I come straight to theatre. The decisions have been made by the medical officers in the ward or in casualty and then I'm the technician. I get called in to do mostly caesars but also ectopic pregnancies and incomplete abortions and that sort of thing. And complications of pregnancy, cervical tears, vaginal tears that sort of thing, retained placentas, but it's only theatre work (Private GP, Hospital E).*

*They book them. We don't really see the patient. We do the surgeries for them just in terms of logistics, and then they follow up with the aftercare after that (Private GP, Hospital C).*

**Profile and outcomes of caesarean deliveries with private GP contracting**

The majority (71%) of caesarean deliveries across all five hospitals were emergency procedures (supplementary table 1). The most common indication for an emergency caesarean delivery was foetal distress or an abnormal CTG (48%) (supplementary table 2). Amongst elective caesarean deliveries the main indication was a previous caesarean delivery (58%). For 5% of emergency caesarean deliveries and 20% of elective caesarean deliveries the indication was missing from the theatre register. Individual hospital documentation style differed with a higher proportion of elective caesarean deliveries (72%) with a documented indication missing at hospital A, which was serviced only by private GPs, whilst this was less than 30% at the other four hospitals. However, this did not affect the overall CS rate (supplementary table 3).

Adverse outcomes following a caesarean delivery indicated by blood loss >1000mls, maternal death post caesarean delivery and referral after caesarean delivery to a regional hospital were rare across all



For peer review only - http://bmjopen.bmj.com/site/about/guidelines.xhtml

hospitals with a 1% prevalence of PPH and 4% of women being transferred to a regional hospital following caesarean delivery. Over the 12-month period of data collection there were no emergency procedures (hysterectomy, B-lynch suture or re-look laparotomies) performed during or following a caesarean delivery at any of the hospitals (supplementary table 4).

Figure 2 shows the utilisation of private GPs for caesarean deliveries and the profile and outcomes of these deliveries. Slightly over half of all caesarean deliveries were performed during public sector working hours (8am-4pm) but this differed across the hospitals. In hospitals A and B the majority of caesarean deliveries were performed during evening / night time (4pm – 8am). In these two hospitals almost all of the caesarean deliveries are performed by private GPs who consult in their private practices during the day. The challenge of balancing a private GP practice with contractual commitments to the district hospital (theme 3) was explained by the following participants:

*So, we come to hospital from eight to ten in the morning. Sometimes it does take longer and you only get down there at half past ten. So, from ten to twelve it's like private practice, then clinics, and then from half past two to half past four, five-ish at the practice again* (private GP, Hospital B).

*If it was a GP that was covering, they would be a bit reluctant to come in at four to assist us because having their own practice, they might still have practice till five or maybe six sometimes. So, they would maybe push the hour that they would be available to come in, a little bit further on, but, usually, if we call for a caesar, it's usually a foetal distress, we don't just cut to cut if it's not a foetal distress then we would say we could wait another half an hour (medical officer, hospital E).*

There is no noticeable increase in elective caesarean deliveries with a higher proportion of total caesarean deliveries undertaken by private GPs and rates of referral to a regional hospital following caesarean delivery was low irrespective of the utilisation of private GPs.

The funding models used to remunerate private GPs for their work within the public sector is a rate per shift irrespective of the number or type of procedures performed. Several GPs described the remuneration model during interviews (theme 4):

*I do one week per month from Monday night, four p.m. till eight a.m. the next Monday and the remuneration is not affected by the number of call outs. It's a flat rate. Whether I get called once in that week or twenty times in that week, the remuneration is the same. Payment every month exactly the same. If I'm lucky, I get called out only once or twice for the week, but, a week ago, I was called out three times in*



For peer review only - http://bmjopen.bmj.com/site/about/guidelines.xhtml

*one night. An ectopic pregnancy, abruptio and, obstructed labour. Just like that. It's four hours in theatre (private GP, Hospital E).*

*You never know how your hours are going to turn out for that week. So, say this week I have a very good week, I only have to be there from eight till eleven. They never phoned me during the night; they never phoned me during my day, the guy who's on next week they're phoning the whole time because it's a caesarean after caesarean. Every night there is an ectopic or a third-degree tear or something so you can't really, you can't really compare the two calls, but everyone still gets the same fixed rate (Private GP, Hospital C).*

*Because the sessional guys (private GPs) are just for the sessions. So, I mean they don't get paid extra if they do ten or one (caesarean deliveries) (Clinical manager, hospital D).*

**Discussion**

This health systems research study explored the utilisation of private GPs to provide caesarean delivery services in rural district hospitals in the Western Cape. We found that the extent of utilisation of private GPs was inversely related to the size of hospital and number of deliveries. The two larger hospitals with over 100 monthly deliveries had a critical mass of doctors to cover the roster and used private GPs less frequently.

In terms of the delivery profile, we found an overall caesarean delivery rate of 21%. This is higher than the WHO recommended rate of 5-15% of all births[16] but lower than the public sector rate for the Western Cape of 29%.[2] Caesarean delivery rates in district hospitals are lower than the national rate because such hospitals refer women with complications such as two previous caesarean deliveries, pre-eclampsia, placenta praevia, etc. to regional or tertiary hospitals. Also, they deliver low risk women having normal vaginal deliveries because they do not have surrounding 24-hour midwife units, so these low-risk births are included in the denominator. Hospital D's caesarean delivery rate (14%) may be lower because the service was closed for several years following an extensive fire in March 2017, with all caesarean deliveries being done elsewhere. The caesarean delivery service resumed again in February 2021 just prior to the start of data collection for this study. This hospital is also unique in being the only one of the five with specialist family physicians overseeing the clinical services. All caesarean deliveries are discussed 24/7 with a family physician prior to being performed, unless such a discussion may delay safe delivery, which may also contribute to the lower rate of caesarean deliveries.



For peer review only - http://bmjopen.bmj.com/site/about/guidelines.xhtml

We did not find an increased caesarean delivery rate in hospitals where a high proportion of caesarean deliveries were performed by private GPs compared to government employed generalist doctors and no increase in caesarean deliveries performed for elective indications. The public-private partnership arrangements in these hospitals are such that the decision-making regarding the need for a caesarean delivery is undertaken by a hospital employee (doctor or midwife) and the private GP is notified once the decision has been made. However, we did find a higher proportion of elective caesarean deliveries without stated indication and a higher proportion performed after 4pm (during evenings and at night) at hospitals more reliant on private GPs. Balancing time between private practice and public sector work may pose a risk to public hospitals where there are delays in private GP availability in emergency situations.

The remuneration model for GP contracting, with a fixed rate irrespective of number of caesarean deliveries should disincentivize over-servicing. A public-private partnership programme in Gujarat state, India provides private obstetricians with a fixed sum per 100 births to provide care to eligible women below the poverty line. The remuneration model was specifically chosen as a disincentive to prevent unnecessary caesarean deliveries.[17] By comparison in Chile, where women covered under public health insurance can choose to give birth within a selected group of private health facilities the caesarean delivery rate was found to be 71% compared to 26% amongst publicly insured women giving birth in public health facilities. Publicly insured women choosing to give birth in a private facility receive a voucher that covers the costs of the delivery except for a 25% co-payment, the sum of which is dependent on the mode of delivery, which is the responsibility of the patient.[18]

In terms of caesarean delivery outcomes our results suggest that in rural district hospitals utilising experienced private GPs to perform caesarean deliveries there was no trend towards increased adverse outcomes. The rate of complications post caesarean section was low across all five hospitals which is an indication of safe caesarean section deliveries overall in this setting.

There was one maternal death following caesarean delivery in the one-year period of review due to eclampsia, a 1% rate of PPH at caesarean delivery and a very low rate of referral to a regional hospital following caesarean delivery. The rate of PPH at caesarean delivery was low. This is likely to reflect the lower risk caesarean deliveries that were performed at district hospitals, good labour ward management with early recourse to caesarean delivery, and surgical skills. It is possible that some women were referred to higher level hospitals for bleeding after caesarean delivery, but this information is not



For peer review only - http://bmjopen.bmj.com/site/about/guidelines.xhtml

available because individual folders were not reviewed, and the numbers would be few because overall referral numbers were low.

In SA, district hospitals account for 38% of public sector caesarean deliveries and the case fatality rate for caesarean deliveries at this level of care is 62 per 100,000 caesarean deliveries.[2] Since these hospitals do not have specialist obstetricians or anaesthetists, surgical and anaesthetic skills training and supervision is critical. Deaths due to bleeding at or following a caesarean delivery have been increasing in SA and lack of skilled doctors has been identified as an important avoidable factor.[19] This model of a public-private partnership makes an important contribution to skills development in rural district hospitals through drawing on the skills and experience of private GPs, and in some hospitals private family physicians, to perform caesarean deliveries alongside hospital-based medical officers many of whom are undertaking community service or recently qualified.

Balancing access with safety is a critical factor in obstetric care. Ideally facilities that can provide care for uncomplicated births should be close to women's homes and within reasonable distance of a district hospital that can perform caesarean deliveries. For small district hospitals with a low monthly delivery rate, maintaining skills to deal with emergencies is a challenge. To be safe and cost-effective, it has been recommended that district hospitals would need to perform between 500 and 1200 deliveries per year.[20] There should also always be a minimum of two doctors on call (one for anaesthesia and one for surgery), so that caesarean deliveries may be performed on a 24-hours-a-day, 7-days-a-week basis.[20] Hospitals A and B in this study performed less than 500 deliveries per year and had the highest utilisation of private GPs.  It would be more costly for these hospitals to employ permanent doctors on the establishment and this model of public private contracting makes financial sense and provides access for women since the nearest regional hospital is roughly 150km from the district.

Considering broader implications of these findings for universal health care it is clear that experienced private GPs can play an important role in contributing much needed surgical and anaesthetic skills for safe caesarean deliveries in rural district hospitals. This is a model that could be considered for other provinces in SA and other low-and-middle income countries provided clinical decisions are driven by accepted public sector protocols and available support from specialists at referral regional hospitals can be ensured. Public-private partnerships have been criticized for being a more urban context option, but this is often on the assumption that the private providers will be obstetric specialists and this is the model that has been applied in settings such as India.[9 10 21] By contrast, in African countries such as Mozambique and Tanzania, clinical officers (healthcare providers trained to perform tasks usually



For peer review only - http://bmjopen.bmj.com/site/about/guidelines.xhtml

undertaken by doctors) have been trained to perform caesarean deliveries. A meta-analysis reported no significant differences in outcomes of caesarean deliveries performed by clinical officers compared to medical doctors.[22] Different approaches to enable access to safe caesarean delivery are therefore needed for different contexts and experienced private GPs are a resource for rural district hospitals to consider. The sustainability of this model depends on the surgical and anaesthetic skills of future generations of GPs. The GPs skilled in obstetric surgery and anaesthesia in rural districts are mostly of an older age many of whom were previously district surgeons (a historical model of contracting private GPs within rural districts to provide curative primary care services within public sector health facilities).[23] More recently qualified GPs may have similar skills to their public sector medical officer equivalents. The scale-up of this model therefore has implications for under-graduate basic surgical and anaesthetics skills training programmes.

**Limitations**

We collected obstetric data from registers and did not perform individual patient folder reviews. The proportion of caesarean delivery complications could have been higher if individual folders were reviewed. Post caesarean sepsis, thromboembolism, or postoperative bleeding that was detected in the postnatal ward, for example, could not be identified with the study methodology. Where no indication was listed in the theatre register for a caesarean delivery, this information would have been found had individual folders been reviewed. Furthermore, although a low proportion of women were referred following caesarean delivery, this group could have experienced morbidity or mortality but were not followed up to determine the reasons for referral or their outcomes at the regional referral hospital. We also did not collect data on neonatal outcomes which is an important indicator of the quality and safety of caesarean delivery.

**Conclusion**

The findings of this study suggest that private GPs can play an important role in filling gaps and expanding quality care in rural public facilities that have insufficient obstetric skills and expertise. In addition, provided that the broader systems and institutional framework to ensure compliance with clinical guidelines and protocols are in place, this can be done in a safe and effective manner, and could be seen as a prototype for NHI in rural contexts.



For peer review only - http://bmjopen.bmj.com/site/about/guidelines.xhtml

**Figure legends**

Figure 1: Private GP utilisation and caesarean delivery rate

Figure 2: Utilisation of private GPs and caesarean section profile and outcomes

**Acknowledgements** We thank all the health professionals who took time to participate in our interviews and to the hospital managers for facilitating access to the obstetric registers. We also thank Vishal Brijlal for his comments on a draft of this manuscript.

**Contributors** TD, GS, ED and SF conceptualised the study; TD, GS, ED and YB analysed the data; TD, GS, ED, AH, SF and YB contributed to the interpretation of the data, writing the paper and reviewing the content.

**Funding** The study was funded by the Bill and Melinda Gates Foundation (Grant Agreement ID INV-023276)

**Competing interests** None declared.
**Patient consent for publication** Not applicable.

**Ethics approval** Ethics approval was granted by the South African Medical Research Council Human Research Ethics Committee (approval number EC003-2/2021) and by the Western Cape Provincial Department of Health and Wellness (approval number WC_202103_017).

**Data availability** The aggregated routine data are available on request to the corresponding author.

For peer review only



For peer review only - http://bmjopen.bmj.com/site/about/guidelines.xhtml

**References**


1. World Health Organisation. Trends in maternal mortality 2000-2017. Geneva: World Health Organisation, 2019.
2. National Department of Health. Saving Mothers 2017-2019. Seventh report on confidential enquiries into maternal deaths in South Africa. Pretoria: National Department of Health, 2020.
3. Solanki G, Fawcus S, Daviaud E. A cross sectional analytic study of modes of delivery and caesarean section rates in a private health insured South African population. *PloS one* 2019;14(6):e0219020. doi: 10.1371/journal.pone.0219020
4. Hoxha I, Fink G. Caesarean sections and health financing: a global analysis. *BMJ Open* 2021;11(5):e044383. doi: 10.1136/bmjopen-2020-044383 [published Online First: 2021/05/26]
5. Sobhy S, Arroyo-Manzano D, Murugesu N, et al. Maternal and perinatal mortality and complications associated with caesarean section in low-income and middle-income countries: a systematic review and meta-analysis. *The Lancet* 2019;393(10184):1973-82. doi: 10.1016/S0140-6736(18)32386-9
6. National Department of Health. Saving Mothers 2014-2016: Seventh triennial report on confidential enquiries into maternal deaths in South Africa. Pretoria: National Department of Health, 2017.
7. National Department of Health. National Health Insurance Bill. In: Health NDo, ed. Pretoria, 2019.
8. Amu H, Dickson KS, Kumi-Kyereme A, et al. Understanding variations in health insurance coverage in Ghana, Kenya, Nigeria, and Tanzania: Evidence from demographic and health surveys. *PloS one* 2018;13(8):e0201833. doi: 10.1371/journal.pone.0201833
9. Iyer V, Sidney K, Mehta R, et al. Availability and provision of emergency obstetric care under a public–private partnership in three districts of Gujarat, India: lessons for Universal Health Coverage. *BMJ Global Health* 2016;1(1):e000019. doi: 10.1136/bmjgh-2015-000019
10. Chaturvedi S, Randive B. Public private partnerships for emergency obstetric care: Lessons from Maharashtra. *Indian Journal of Community Medicine* 2011;36(1):21-26. doi: 10.4103/0970-0218.80788
11. Creswell John W, Plano Clark Vicki L. Choosing a Mixed Methods Design. Designing and Conducting Mixed Methods Research. 2 ed. Los Angeles, London, New Dehli: Sage 2011:53-106.
12. Green J, Thorogood, N. Qualitative Methods for Health Research. London: Sage Publications 2004.
13. Department of Health. A District Hospital Service Package for South Africa. Pretoria: Department of Health, 2002.
14. Gale NK, Heath G, Cameron E, et al. Using the framework method for the analysis of qualitative data in multi-disciplinary health research. *BMC Med Res Methodol* 2013;13:117. doi: 10.1186/1471-2288-13-117
15. Solanki GC, Cornell JE, Daviaud E, et al. Caesarean section rates in South Africa: A case study of the health systems challenges for the proposed National Health Insurance. *SAMJ* 2020;110(8)
16. World Health Organisation. WHO statement on caesarean section rates. Geneva: WHO, 2015.
17. Salazar M, Vora K, Sidney Annerstedt K, et al. Caesarean sections in the in the context of the Chiranjeevi Yojana public private partnership program to promote institutional birth in Gujarat, India; does the embedded disincentive for caesarean section work? *International journal for equity in health* 2019;18(1):17-17. doi: 10.1186/s12939-019-0922-5
18. Borrescio-Higa F, Valdés N. Publicly insured caesarean sections in private hospitals: a repeated cross-sectional analysis in Chile. *BMJ open* 2019;9(4):e024241-e41. doi: 10.1136/bmjopen-2018-024241
19. Fawcus S, Pattinson RC, Moodley J, et al. Maternal deaths from bleeding associated with caesarean delivery: A national emergency. *SAMJ* 2016;106(5)
20. Pattinson RC. Safety versus accessibility in maternal and perinatal care. *SAMJ* 2015;105(4)




For peer review only - http://bmjopen.bmj.com/site/about/guidelines.xhtml

21. Randive B, Chaturvedi S, Mistry N. Contracting in specialists for emergency obstetric care- does it work in rural India? *BMC Health Services Research* 2012;12(1):485. doi: 10.1186/1472-6963-12-485
22. Wilson A, Lissauer D, Thangaratinam S, et al. A comparison of clinical officers with medical doctors on outcomes of caesarean section in the developing world: meta-analysis of controlled studies. *Bmj* 2011;342:d2600. doi: 10.1136/bmj.d2600 [published Online First: 2011/05/17]
23. Kula N, Fryatt RJ. Public–private interactions on health in South Africa: opportunities for scaling up. *Health Policy and Planning* 2013;29(5):560-69. doi: 10.1093/heapol/czt042

For peer review only



For peer review only - http://bmjopen.bmj.com/site/about/guidelines.xhtml

Figure 1: Private GP utilisation and caesarean delivery rate

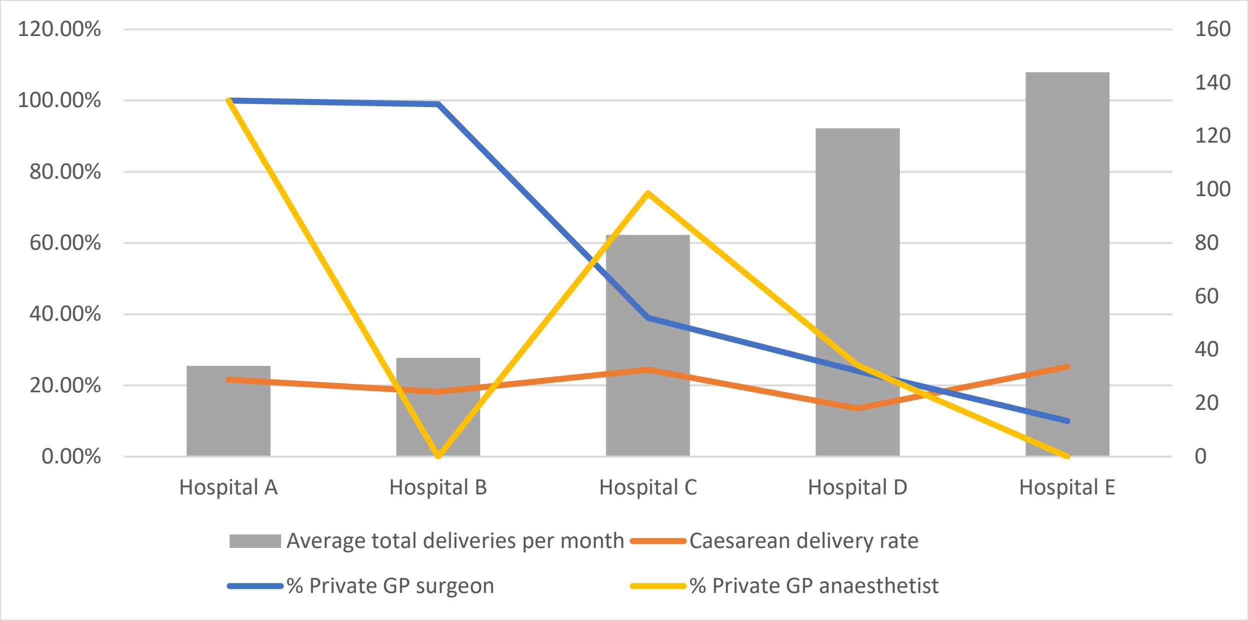

Figure 2: Utilisation of private GPs and caesarean delivery profile and outcomes

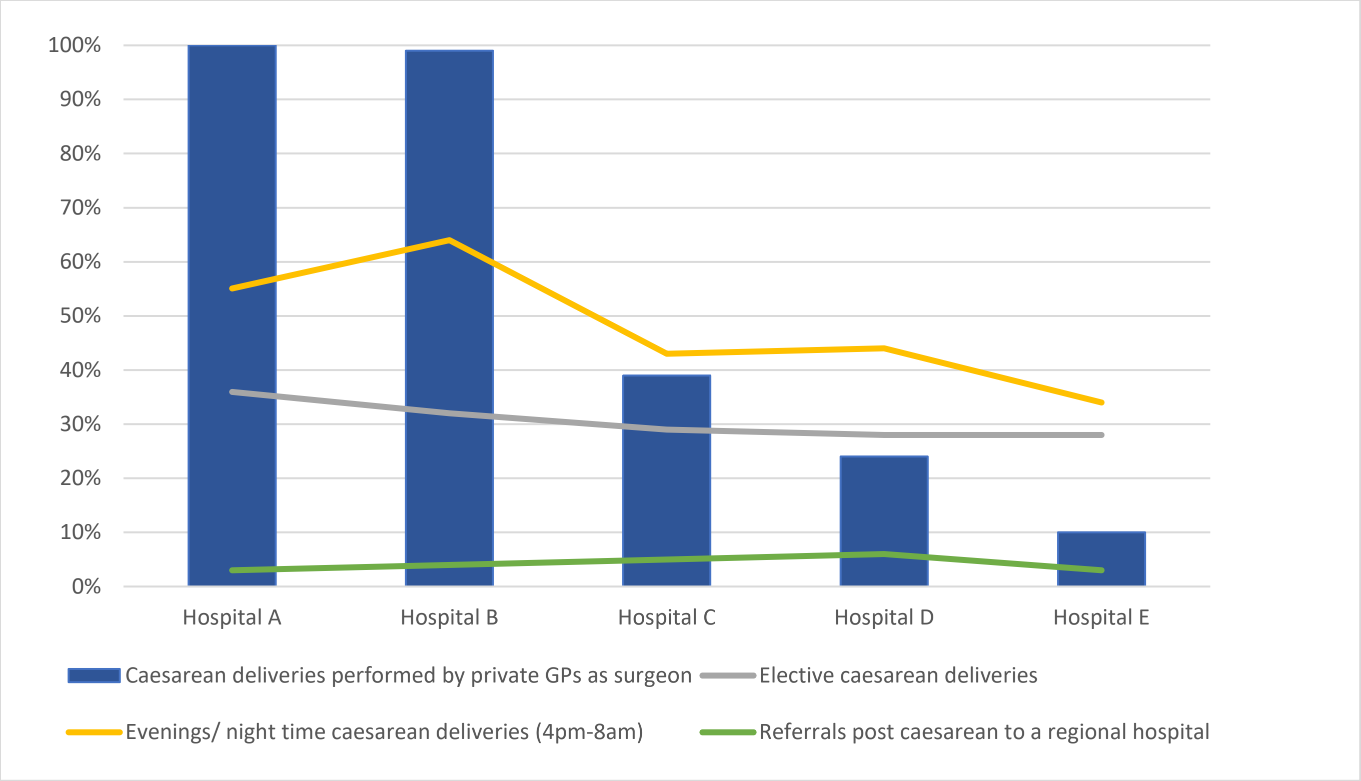

Supplementary tables

Supplementary table 1: Summary of deliveries and private GP utilisation 01 April 2021 – 31 March 2022

| | Hospital A | | Hospital B | | Hospital C | | Hospital D | | Hospital E | | All Hospitals | |
|---|---|---|---|---|---|---|---|---|---|---|---|---|
| **Delivery profile** | **N** | **%** | **N** | **%** | **N** | **%** | **N** | **%** | **N** | **%** | **N** | **%** |
| Normal vaginal delivery (NVD) | 311 | 75 | 356 | 80 | 732 | 74 | 1,259 | 85 | 1,271 | 74 | 3,929 | 78 |
| Assisted vaginal (vacuum or forceps) | 12 | 3 | 9 | 2 | 17 | 2 | 13 | 1 | 21 | 1 | 72 | 1 |
| Caesarean deliveries | 89 | 22 | 81 | 18 | 242 | 24 | 199 | 14 | 436 | 25 | 1,047 | 21 |
| **Total** | **412** | | **446** | | **991** | | **1,471** | | **1,728** | | **5,048** | |
| **Private GP utilisation for caesarean deliveries** | | | | | | | | | | | | |
| **Surgeon** | | | | | | | | | | | | |
| Public Sector non specialist Surgeon | 0 | 0 | 1 | 1 | 148 | 61 | 152 | 76 | 394 | 90 | 706 | 67 |
| Private GP Surgeon | 89 | 100 | 80 | 99 | 94 | 39 | 47 | 24 | 42 | 10 | 341 | 33 |
| **Anaesthetist** | | | | | | | | | | | | |
| Public sector non specialist anaesthetist | 0 | 0 | 81 | 100 | 63 | 26 | 148 | 74 | 436 | 100 | 746 | 71 |
| Private GP Anaesthetist | 89 | 100 | 0 | 0 | 179 | 74 | 51 | 26 | 0 | 0 | 300 | 29 |
| **Profile of caesarean deliveries** | | | | | | | | | | | | |
| **Hours, Monday-Sunday:** | | | | | | | | | | | | |
| Evening/ night-time (4pm-8am) | 49 | 55 | 52 | 64 | 103 | 43 | 88 | 44 | 150 | 34 | 442 | 42 |
| Daytime hours (8am-4pm) | 40 | 45 | 29 | 36 | 139 | 57 | 111 | 56 | 286 | 66 | 605 | 58 |
| **Elective vs. Emergency** | | | | | | | | | | | | |
| Elective caesarean | 32 | 36 | 26 | 32 | 71 | 29 | 56 | 28 | 121 | 28 | 306 | 29 |
| Emergency caesarean | 57 | 64 | 55 | 68 | 171 | 71 | 143 | 72 | 316 | 72 | 742 | 71 |
| **Anaesthetic Type** | | | | | | | | | | | | |
| Spinal | 86 | 97 | 79 | 99 | 218 | 92 | 192 | 96 | 414 | 95 | 989 | 95 |
| General anaesthetic | 3 | 3 | 1 | 1 | 16 | 7 | 5 | 3 | 20 | 4.5 | 45 | 4 |
| Combined spinal and general | 0 | 0 | 0 | 0 | 3 | 1 | 2 | 1 | 2 | 0.5 | 7 | 1 |

Supplementary table 2: Indications for emergency caesarean deliveries

| Indications * | Hospital A | | Hospital B | | Hospital C | | Hospital D | | Hospital E | | All Hospitals | |
|---|---|---|---|---|---|---|---|---|---|---|---|---|
| | N | % | N | % | N | % | N | % | N | % | N | % |
| Fetal distress/abnormal CTG | 15 | 26 | 28 | 51 | 77 | 45 | 79 | 55 | 155 | 49 | 354 | 48 |
| Prolonged labour/CPD | 6 | 10 | 10 | 18 | 24 | 14 | 18 | 12 | 37 | 12 | 95 | 13 |
| Previous CD (in labour) | 4 | 7 | 3 | 5 | 42 | 25 | 9 | 6 | 12 | 4 | 70 | 9 |
| Malpresentation (in labour) | 12 | 21 | 6 | 11 | 11 | 6 | 11 | 8 | 30 | 10 | 70 | 9 |
| Failed induction | 2 | 4 | 1 | 2 | 2 | 1 | 8 | 6 | 27 | 8 | 40 | 5 |
| placental abruption | 2 | 4 | 1 | 2 | 5 | 3 | 6 | 4 | 3 | 1 | 17 | 2 |
| Pre-eclampsia | 0 | 0 | 1 | 2 | 0 | 0 | 4 | 3 | 9 | 3 | 14 | 2 |
| PROM | 3 | 5 | 0 | 0 | 0 | 0 | 0 | 0 | 3 | 1 | 6 | 1 |
| Multiple pregnancy | 1 | 2 | 2 | 4 | 0 | 0 | 2 | 1 | 0 | 0 | 5 | 1 |
| APH | 0 | 0 | 0 | 0 | 0 | 0 | 1 | 1 | 2 | 1 | 3 | 0.4 |
| Failed VBAC | 0 | 0 | 0 | 0 | 0 | 0 | 0 | 0 | 0 | 0 | 0 | 0 |
| Big baby | 0 | 0 | 0 | 0 | 0 | 0 | 0 | 0 | 0 | 0 | 0 | 0 |
| Other | 2 | 4 | 3 | 5 | 4 | 2 | 4 | 3 | 17 | 5 | 30 | 4 |
| Number without listed indication | 10 | 17 | 0 | 0 | 6 | 4 | 1 | 1 | 20 | 6 | 37 | 5 |
| **Grand Total** | **57** | **100.00%** | **55** | **100.00%** | **171** | **100.00%** | **143** | **100.00%** | **315** | **100.00%** | **741** | **100.00%** |

CTG = cardiotocography ; CPD= cephalopelvic disproportion; APH = antepartum haemorrhage; CD = caesarean delivery; VBAC = vaginal birth after Caesarean;

PROM = premature rupture of membranes; APH = antepartum haemorrhage

*The indications for caesarean deliveries were those that were written in the theatre register so it was not possible to interrogate some of the indications such as PROM (prolonged rupture of membranes). According to Western Cape guidelines, caesarean delivery can be performed at District hospitals for suspected Big Baby. However, VBACs are not routinely performed at District hospitals and patients with pre-eclampsia would all be referred to regional or tertiary hospitals

For peer review only - http://bmjopen.bmj.com/site/about/guidelines.xhtml

Supplementary table 3: indications for elective caesarean sections

| Indications* | Hospital A | | Hospital B | | Hospital C | | Hospital D | | Hospital E | | All Hospitals | |
|---|---|---|---|---|---|---|---|---|---|---|---|---|
| | N | % | N | % | N | % | N | % | N | % | N | % |
| Previous CD | 5 | 16 | 16 | 61 | 50 | 70 | 40 | 71 | 66 | 55 | 177 | 58 |
| Malpresentation (breech/transverse) | 2 | 6 | 0 | 0 | 4 | 6 | 5 | 9 | 13 | 11 | 24 | 8 |
| HIV & unsuppressed VL | 1 | 3 | 2 | 8 | 0 | 0 | 0 | 0 | 8 | 7 | 11 | 3 |
| Multiple pregnancy | 0 | 0 | 0 | 0 | 1 | 1.4 | 1 | 2 | 3 | 2 | 5 | 2 |
| Big baby | 0 | 0 | 0 | 0 | 1 | 1.4 | 0 | 0 | 3 | 2 | 4 | 1 |
| Patient request | 0 | 0 | 0 | 0 | 0 | 0 | 0 | 0 | 2 | 2 | 2 | 1 |
| Diabetes & macrosomia | 0 | 0 | 0 | 0 | 0 | 0 | 0 | 0 | 1 | 1 | 1 | 0.3 |
| Obstructive vulvar warts | 0 | 0 | 0 | 0 | 0 | 0 | 0 | 0 | 0 | 0 | 0 | 0 |
| Other (genital herpes etc) | 1 | 3 | 1 | 4 | 7 | 10 | 1 | 2 | 10 | 8 | 20 | 7 |
| Number without listed indication | 23 | 72 | 7 | 27 | 8 | 11 | 9 | 16 | 15 | 12 | 62 | 20 |
| **Grand Total** | **32** | **100.00%** | **26** | **100.00%** | **71** | **100.00%** | **56** | **100.00%** | **121** | **100.00%** | **306** | **100.00%** |

CD = caesarean delivery; VL = viral load

*The indications for caesarean deliveries were those that were written in the theatre register so it was not possible to interrogate some of the indications. According to Western Cape guidelines, caesarean delivery can be performed at District hospitals for suspected Big Baby.

For peer review only

Supplementary table 4: Adverse outcomes following caesarean deliveries

| | Hospital A | | Hospital B | | Hospital C | | Hospital D | | Hospital E | | All Hospitals | |
|---|---|---|---|---|---|---|---|---|---|---|---|---|
| | N | % | N | % | N | % | N | % | N | % | N | % |
| **Adverse Outcomes** | | | | | | | | | | | | |
| Total PPH/estimated blood loss >=1000 mls | 0 | 0 | 1 | 1 | 2 | 1 | 0 | 0 | 10 | 2 | 13 | 1 |
| Maternal deaths post caesarean | 0 | 0 | 0 | 0 | 1* | 0.4 | 0 | 0 | 0 | 0 | 1 | 0.1 |
| Referrals post caesarean to a regional hospital | 3 | 3 | 3 | 4 | 12 | 5 | 11 | 6 | 11 | 3 | 40 | 4 |
| **Procedures performed at caesarean deliveries** | | | | | | | | | | | | |
| IUCD/tubal ligation | 13 | 15 | 5 | 6 | 33 | 14 | 16 | 8 | 43 | 10 | 110 | 11 |
| Other Procedures | 0 | 0 | 0 | 0 | 0 | 0 | 5 | 3 | o | 0 | 5 | 0.5 |
| Hysterectomies at/after caesarean | 0 | 0 | 0 | 0 | 0 | 0 | 0 | 0 | 0 | 0 | 0 | 0 |
| B lynch suture/balloon tamponade | 0 | 0 | 0 | 0 | 0 | 0 | 0 | 0 | 0 | 0 | 0 | 0 |
| Relook laparotomies | 0 | 0 | 0 | 0 | 0 | 0 | 0 | 0 | 0 | 0 | 0 | 0 |

PPH = postpartum haemorrhage; IUCD = intrauterine contraceptive device * cause of death was eclampsia

For peer review only

# Standards for Reporting Qualitative Research (SRQR)*

http://www.equator-network.org/reporting-guidelines/srqr/

## Title and abstract

| | Page/line no(s). |
|---|---|
| **Title** - Concise description of the nature and topic of the study Identifying the study as qualitative or indicating the approach (e.g., ethnography, grounded theory) or data collection methods (e.g., interview, focus group) is recommended | 1 |
| **Abstract** - Summary of key elements of the study using the abstract format of the intended publication; typically includes background, purpose, methods, results, and conclusions | 2 |

## Introduction

| | |
|---|---|
| **Problem formulation** - Description and significance of the problem/phenomenon studied; review of relevant theory and empirical work; problem statement | 5 |
| **Purpose or research question** - Purpose of the study and specific objectives or questions | 5 |

## Methods

| | |
|---|---|
| **Qualitative approach and research paradigm** - Qualitative approach (e.g., ethnography, grounded theory, case study, phenomenology, narrative research) and guiding theory if appropriate; identifying the research paradigm (e.g., postpositivist, constructivist/ interpretivist) is also recommended; rationale** | 6 |
| **Researcher characteristics and reflexivity** - Researchers' characteristics that may influence the research, including personal attributes, qualifications/experience, relationship with participants, assumptions, and/or presuppositions; potential or actual interaction between researchers' characteristics and the research questions, approach, methods, results, and/or transferability | 8 |
| **Context** - Setting/site and salient contextual factors; rationale** | 6/7 |
| **Sampling strategy** - How and why research participants, documents, or events were selected; criteria for deciding when no further sampling was necessary (e.g., sampling saturation); rationale** | 8 |
| **Ethical issues pertaining to human subjects** - Documentation of approval by an appropriate ethics review board and participant consent, or explanation for lack thereof; other confidentiality and data security issues | 9/10 |
| **Data collection methods** - Types of data collected; details of data collection procedures including (as appropriate) start and stop dates of data collection and analysis, iterative process, triangulation of sources/methods, and modification of procedures in response to evolving study findings; rationale** | 8 |



For peer review only - http://bmjopen.bmj.com/site/about/guidelines.xhtml

| | |
|---|---|
| **Data collection instruments and technologies** - Description of instruments (e.g., interview guides, questionnaires) and devices (e.g., audio recorders) used for data collection; if/how the instrument(s) changed over the course of the study | 8 |
| **Units of study** - Number and relevant characteristics of participants, documents, or events included in the study; level of participation (could be reported in results) | 8 |
| **Data processing** - Methods for processing data prior to and during analysis, including transcription, data entry, data management and security, verification of data integrity, data coding, and anonymization/de-identification of excerpts | 9 |
| **Data analysis** - Process by which inferences, themes, etc., were identified and developed, including the researchers involved in data analysis; usually references a specific paradigm or approach; rationale** | 9 |
| **Techniques to enhance trustworthiness** - Techniques to enhance trustworthiness and credibility of data analysis (e.g., member checking, audit trail, triangulation); rationale** | 9 |

**Results/findings**

| | |
|---|---|
| **Synthesis and interpretation** - Main findings (e.g., interpretations, inferences, and themes); might include development of a theory or model, or integration with prior research or theory | 10-17 |
| **Links to empirical data** - Evidence (e.g., quotes, field notes, text excerpts, photographs) to substantiate analytic findings | 10-17 |

**Discussion**

| | |
|---|---|
| **Integration with prior work, implications, transferability, and contribution(s) to the field** - Short summary of main findings; explanation of how findings and conclusions connect to, support, elaborate on, or challenge conclusions of earlier scholarship; discussion of scope of application/generalizability; identification of unique contribution(s) to scholarship in a discipline or field | 18-20 |
| **Limitations** - Trustworthiness and limitations of findings | 21 |

**Other**

| | |
|---|---|
| **Conflicts of interest** - Potential sources of influence or perceived influence on study conduct and conclusions; how these were managed | 22 |
| **Funding** - Sources of funding and other support; role of funders in data collection, interpretation, and reporting | 22 |

*The authors created the SRQR by searching the literature to identify guidelines, reporting standards, and critical appraisal criteria for qualitative research; reviewing the reference lists of retrieved sources; and contacting experts to gain feedback. The SRQR aims to improve the transparency of all aspects of qualitative research by providing clear standards for reporting qualitative research.



For peer review only

For peer review only - http://bmjopen.bmj.com/site/about/guidelines.xhtml

**The rationale should briefly discuss the justification for choosing that theory, approach, method, or technique rather than other options available, the assumptions and limitations implicit in those choices, and how those choices influence study conclusions and transferability. As appropriate, the rationale for several items might be discussed together.

**Reference:**

O'Brien BC, Harris IB, Beckman TJ, Reed DA, Cook DA. **Standards for reporting qualitative research: a synthesis of recommendations.** *Academic Medicine*, Vol. 89, No. 9 / Sept 2014 DOI: 10.1097/ACM.0000000000000388

For peer review only



For peer review only - http://bmjopen.bmj.com/site/about/guidelines.xhtml

STROBE Statement—checklist of items that should be included in reports of observational studies

| | Item No. | Recommendation | Page No. | Relevant text from manuscript |
|---|---|---|---|---|
| **Title and abstract** | 1 | (*a*) Indicate the study's design with a commonly used term in the title or the abstract | 1 | |
| | | (*b*) Provide in the abstract an informative and balanced summary of what was done and what was found | 2 | |
| **Introduction** | | | | |
| Background/rationale | 2 | Explain the scientific background and rationale for the investigation being reported | 4&5 | |
| Objectives | 3 | State specific objectives, including any prespecified hypotheses | 5 | |
| **Methods** | | | | |
| Study design | 4 | Present key elements of study design early in the paper | 6&7 | |
| Setting | 5 | Describe the setting, locations, and relevant dates, including periods of recruitment, exposure, follow-up, and data collection | 6&7 | |
| Participants | 6 | (*a*) *Cohort study*—Give the eligibility criteria, and the sources and methods of selection of participants. Describe methods of follow-up<br>*Case-control study*—Give the eligibility criteria, and the sources and methods of case ascertainment and control selection. Give the rationale for the choice of cases and controls<br>*Cross-sectional study*—Give the eligibility criteria, and the sources and methods of selection of participants | 7 | |
| | | (*b*) *Cohort study*—For matched studies, give matching criteria and number of exposed and unexposed<br>*Case-control study*—For matched studies, give matching criteria and the number of controls per case | | |
| Variables | 7 | Clearly define all outcomes, exposures, predictors, potential confounders, and effect modifiers. Give diagnostic criteria, if applicable | 7 | |
| Data sources/ measurement | 8* | For each variable of interest, give sources of data and details of methods of assessment (measurement). Describe comparability of assessment methods if there is more than one group | 7 | |
| Bias | 9 | Describe any efforts to address potential sources of bias | 21 | |
| Study size | 10 | Explain how the study size was arrived at | 7 | |

Continued on next page



For peer review only

| | | | |
|---|---|---|---|
| Quantitative variables | 11 | Explain how quantitative variables were handled in the analyses. If applicable, describe which groupings were chosen and why | 9 |
| Statistical methods | 12 | (*a*) Describe all statistical methods, including those used to control for confounding | 9 |
| | | (*b*) Describe any methods used to examine subgroups and interactions | 9 |
| | | (*c*) Explain how missing data were addressed | |
| | | (*d*) *Cohort study*—If applicable, explain how loss to follow-up was addressed<br>*Case-control study*—If applicable, explain how matching of cases and controls was addressed<br>*Cross-sectional study*—If applicable, describe analytical methods taking account of sampling strategy | |
| | | (*e*) Describe any sensitivity analyses | |
| **Results** | | | |
| Participants | 13* | (a) Report numbers of individuals at each stage of study—eg numbers potentially eligible, examined for eligibility, confirmed eligible, included in the study, completing follow-up, and analysed | table 2 |
| | | (b) Give reasons for non-participation at each stage | |
| | | (c) Consider use of a flow diagram | |
| Descriptive data | 14* | (a) Give characteristics of study participants (eg demographic, clinical, social) and information on exposures and potential confounders | Table 1 |
| | | (b) Indicate number of participants with missing data for each variable of interest | |
| | | (c) *Cohort study*—Summarise follow-up time (eg, average and total amount) | |
| Outcome data | 15* | *Cohort study*—Report numbers of outcome events or summary measures over time | |
| | | *Case-control study*—Report numbers in each exposure category, or summary measures of exposure | |
| | | *Cross-sectional study*—Report numbers of outcome events or summary measures | tables 2 and 3 |
| Main results | 16 | (*a*) Give unadjusted estimates and, if applicable, confounder-adjusted estimates and their precision (eg, 95% confidence interval). Make clear which confounders were adjusted for and why they were included | pages 10-17 |
| | | (*b*) Report category boundaries when continuous variables were categorized | table 1 |
| | | (*c*) If relevant, consider translating estimates of relative risk into absolute risk for a meaningful time period | |

Continued on next page

For peer review only

| | | | |
|---|---|---|---|
| Other analyses | 17 | Report other analyses done—eg analyses of subgroups and interactions, and sensitivity analyses | |
| **Discussion** | | | |
| Key results | 18 | Summarise key results with reference to study objectives | 18-20 |
| Limitations | 19 | Discuss limitations of the study, taking into account sources of potential bias or imprecision. Discuss both direction and magnitude of any potential bias | 21 |
| Interpretation | 20 | Give a cautious overall interpretation of results considering objectives, limitations, multiplicity of analyses, results from similar studies, and other relevant evidence | 21 |
| Generalisability | 21 | Discuss the generalisability (external validity) of the study results | 21 |
| **Other information** | | | |
| Funding | 22 | Give the source of funding and the role of the funders for the present study and, if applicable, for the original study on which the present article is based | 22 |

*Give information separately for cases and controls in case-control studies and, if applicable, for exposed and unexposed groups in cohort and cross-sectional studies.

**Note:** An Explanation and Elaboration article discusses each checklist item and gives methodological background and published examples of transparent reporting. The STROBE checklist is best used in conjunction with this article (freely available on the Web sites of PLoS Medicine at http://www.plosmedicine.org/, Annals of Internal Medicine at http://www.annals.org/, and Epidemiology at http://www.epidem.com/). Information on the STROBE Initiative is available at www.strobe-statement.org.

For peer review only
